# Machine learning reveals a multimodal, transdiagnostic signature of emotion dysregulation vulnerability across patients, offspring, and controls

**DOI:** 10.1038/s41380-026-03767-1

**Published:** 2026-07-27

**Authors:** Luigi F. Saccaro, Thomas Larrieu, Farnaz Delavari, Céline Pellaton, Ben Meuleman, Nader Perroud, Dimitri Van De Ville, Nicolas Toni, Camille Piguet

**Affiliations:** 1 Psychiatry Department, Faculty of Medicine, University of Geneva, Geneva, Switzerland; 2 Psychiatry Department, Geneva University Hospital, Geneva, Switzerland; 3 Medical Image Processing Laboratory, Neuro-X Institute, École Polytechnique Fédérale de Lausanne, Geneva, Switzerland; 4 Center for Psychiatric Neuroscience, Department of Psychiatry, Lausanne University Hospital, University of Lausanne, Prilly, Switzerland; 5 Developmental Imaging and Psychopathology Laboratory, University of Geneva School of Medicine, Geneva, Switzerland; 6 Division of Immunology and Allergy, Lausanne University Hospital (CHUV) and University of Lausanne, Lausanne, Switzerland; 7 Swiss Center for Affective Sciences, University of Geneva, Geneva, Switzerland; 8 Department of Radiology and Medical Informatics, Faculty of Medicine, University of Geneva, Geneva, Switzerland; 9 General Pediatric Division, Geneva University Hospital, Geneva, Switzerland

**Keywords:** Predictive markers, Neuroscience, Psychology, ADHD, Bipolar disorder

## Abstract

Emotion dysregulation (ED) is a core transdiagnostic feature of several psychiatric disorders, including borderline personality disorder, bipolar disorder, and attention-deficit/hyperactivity disorder. These ED disorders (EDD) exhibit overlapping clinical presentations, shared heritability, and common neurobiological substrates. This study used a transdiagnostic framework to identify early and multimodal markers of vulnerability, particularly in high-risk populations such as the offspring of EDD patients (EDDoff). A total of 237 participants (97 EDD patients, 67 EDDoff, 73 healthy controls; 139 females in total) completed a multimodal assessment including clinical evaluations, diffusion and functional MRI, and immune and neurotrophic serum biomarkers. Dimensionality reduction was performed using principal component analysis (PCA), and exploratory random forest (RF) models were trained for group classification and symptoms prediction. PCA on the full multimodal dataset yielded eight components, two of which significantly differed between groups, one reflecting high ED and altered hippocampal dynamic functional connectivity (dFC), for which EDDoff showed an intermediate phenotype, and another driven by systemic inflammation, increased in EDD patients only. Modality-specific PCA identified significant inter-modality correlations, including reduced white matter integrity with increasing immune dysregulation, and positive correlations between hippocampal dFC and both ED symptoms and inflammation (*p*_*adj*_ = < 0.01 for all correlations). A RF classifier achieved a balanced accuracy of 80.3% in distinguishing controls from EDD/EDDoff individuals. Multimodal non-clinical features predicted ED symptoms (*p* < 0.01). This study identifies a specific, clinically relevant, transdiagnostic and multimodal signature of vulnerability to ED, spanning behavioral, neural, and immune systems. This multimodal profile may inform future early intervention strategies targeting at-risk populations, such as EDDoff.

## Introduction

Emotion dysregulation (ED) refers to difficulties in modulating emotional experiences and responses in an adaptive, context-appropriate manner [1, 2]. Conceptualized as a core dimension cutting across psychopathology and proposed as a distinct domain within the National Institute of Mental Health’s Research Domain Criteria framework, ED is expressed through affective instability, heightened emotional reactivity, and the use of non-adaptive regulation strategies. Growing evidence indicates that ED is central to several severe and common psychiatric conditions, including borderline personality disorder (BPD) [3], bipolar disorder (BD) [4], and attention-deficit/hyperactivity disorder (ADHD) [2, 5], hereafter collectively referred to as emotion dysregulation disorders (EDD). These disorders show convergent clinical features (e.g. impulsivity, affective lability, interpersonal instability, increased risk of substance abuse, comorbidity with anxiety disorders), substantial shared heritability [6–9], overlapping neurobiological substrates, and high rates of comorbidity [10–14]. This convergence challenges traditional categorical diagnostic frameworks and underscores the need for transdiagnostic and dimensional approaches that prioritize cross-cutting mechanisms and integrate biological, psychological, and behavioral domains to improve risk prediction and intervention development [2, 15].

Despite an increased emphasis on proactive prevention and early identification of at-risk individuals, robust biomarkers that reliably predict vulnerability to psychiatric disorders remain elusive [16, 17]. This challenge may arise not only from the extensive overlap in symptoms [18], genetic influences, and neurobiological characteristics across diagnostic categories [19], but also from the fact that the earlier one attempts to detect vulnerability, the subtler and less pronounced the underlying biological signatures are likely to be. Investigating ED as a shared liability dimension may help address these limitations by providing mechanistically meaningful transdiagnostic targets that transcend current nosological boundaries. In this context, including both patients with established EDD and at-risk subjects, alongside healthy controls, may allow probing a gradient from manifest disorder to risk to low-risk status, thereby facilitating the identification of early, subthreshold markers that are embedded within the broader spectrum of ED. Hence, studying individuals at familial risk, such as offspring of EDD patients (EDDoff), offers a valuable opportunity to identify early ED-related transdiagnostic vulnerability markers that may inform early intervention strategies. Notably, the onset of EDD or ED-related signs often emerges during adolescence and young adulthood, a critical developmental period coinciding with the maturation of emotion regulation fronto-limbic circuits [20]. This developmental period represents a critical window of opportunity for identifying early ED-related vulnerability markers. Given the substantial comorbidity and overlapping features across EDD described below and in the Supplementary Background, there is an urgent need for reliable, transdiagnostic vulnerability markers that can guide early detection, risk stratification, and targeted interventions [21, 22]. Although early interventions have demonstrated promise [22], identifying robust early markers has proven challenging due to the complex, multifactorial nature of EDD risk.

Previous studies have linked ED to early life stress, which promotes a pro-inflammatory state via dysregulation of the hypothalamic-pituitary-adrenal (HPA) axis [23–28], suggesting a pivotal role in the etiology of EDD [23–30]. In addition, stress and inflammatory processes have been shown to disrupt hippocampal structure and function [31–35]. The hippocampus, being part of the prefrontal cortical-hippocampal-amygdala emotion-processing circuit, is essential for integrating emotion, memory, and stress responses [4]. Thus, from a functional brain network perspective, ED is associated with alterations not only in the hippocampus [36–39] and in the rest of the limbic network (LN) as well as ventromedial prefrontal and lateral prefrontal regions [4, 40–43], but also in the sensorimotor network (SMN) [29, 44, 45], which plays a role in both cognitive and emotional processing in EDD [44, 46–57]. Disruption of SMN connectivity, particularly with the hippocampus, may compromise sensorimotor integration and the appraisal of emotional stimuli, thereby contributing to the core features of ED. These network disruptions may exacerbate ED symptoms, thereby perpetuating a vicious cycle of stress, inflammation, and ED (Supplementary Figure 1). Indeed, peripheral inflammation is increasingly recognized as a contributor to central white matter alterations through several plausible mechanisms. Inflammatory cytokines can compromise blood-brain barrier integrity, which may already be impaired in some EDD patients [58], thus facilitating the infiltration of peripheral immune mediators into the central nervous system. These mediators can promote neurotoxic pathways such as the tryptophan-kynurenine axis, impairing oligodendrocyte function and inhibiting remyelination, resulting in microstructural damage [59, 60]. Additionally, inflammatory markers have been identified post-mortem within the hippocampi of EDD patients [61], and immunoglobulins derived from the serum of individuals with EDD have demonstrated the ability to hydrolyze human myelin basic protein in vitro [62]. These findings align with the hypothesis that peripheral immune dysregulation, through mechanisms such as inflammatory infiltration or circulating autoantibodies, may impact neural integrity, including central myelinated pathways implicated in emotion regulation [29, 30, 63, 64].

Despite this growing evidence for shared neurobiological substrates across EDD, and individual studies linking EDD to neural and immune dysfunction [47, 48, 52, 65], to our knowledge, evidence integrating clinical, structural, functional, and immune measures within a transdiagnostic framework, particularly in high-risk populations, remains limited or lacking.

To address this gap, the present study combines clinical symptom assessments, functional and diffusion MRI, and serum biomarkers in a transdiagnostic and multimodal analysis. Using principal component analysis (PCA) for dimensionality reduction and exploratory random forest (RF) machine learning for group classification and symptom prediction, we aim to identify integrated markers of ED vulnerability and expression across EDD patients, EDD patients’ offspring, and healthy controls, spanning a continuum from high to low ED. We hypothesized that both EDD and familial risk for EDD would be associated with alterations in structural and functional brain connectivity and with peripheral immune dysregulation. This integrative approach is designed to uncover convergent signatures of ED, highlight transdiagnostic neural and immune associations, and inform early intervention strategies targeting vulnerable individuals.

## Methods

### Participants

Participants were recruited into three distinct groups, as previously described [66]: adult patients with EDD (including BD, BPD, or ADHD), offspring of other EDD patients (EDDoff), and healthy controls (HC). No exclusion criteria based on age or disease stage were enforced. Detailed exclusion criteria are in the Supplementary Methods. EDD patients fulfilled DSM-IV-TR diagnostic criteria and were recruited from the Mood and ED Clinics at Geneva University Hospitals’ Psychiatry Department. EDDoff were individuals who had at least one parent diagnosed with EDD (BD, BPD, or ADHD). To control for familial confounding factors, none of the participants across groups were biologically related. HC participants were recruited through web-based announcements or local research databases.

**Fig. 1:**
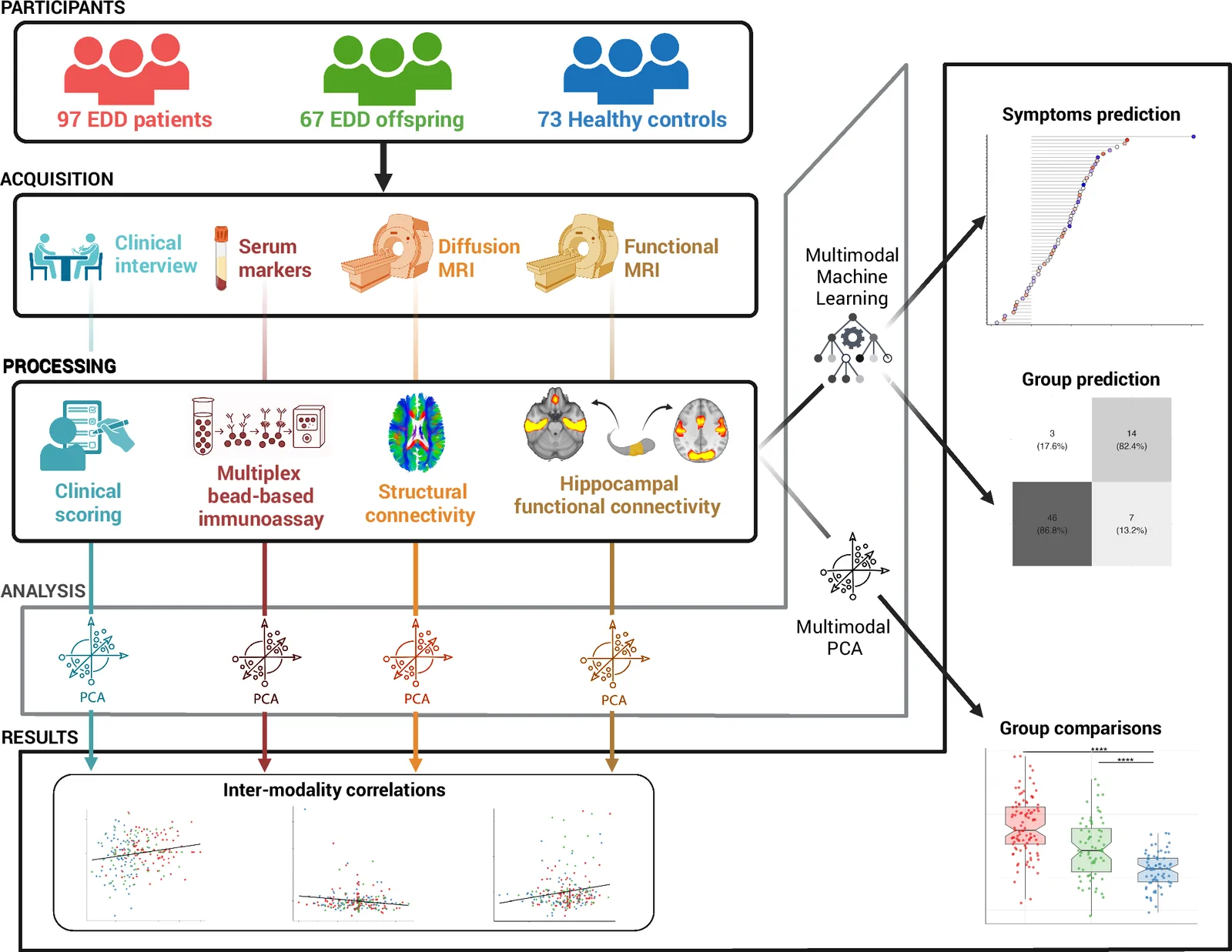
Schematic overview of the study design. A total of 237 participants, 97 with emotion dysregulation disorders (EDD), 67 offspring of EDD patients (EDDoff), and 73 healthy controls, underwent four types of assessment: clinical measures, serum biomarkers, diffusion‐weighted MRI (structural connectivity), and resting‐state functional MRI (hippocampal dynamic functional connectivity). Principal component analysis (PCA) was first applied to the full dataset to extract transdiagnostic components that differ by group, then separately within each modality (block‐level PCA) to examine inter‐modality correlations. Random forest machine learning models used these multimodal features to predict groups and emotion dysregulation symptoms.

Written informed consent was obtained from all participants, as approved by the Geneva University Ethical Committee (CER 13-081). All participants were evaluated using a comprehensive multimodal assessment battery, incorporating clinical assessment, biological markers, and neuroimaging-derived variables, including structural and dynamic functional connectivity (dFC) metrics. Supplementary Table 1 provides a complete list of all variables acquired and corresponding abbreviations. The details for the acquisition of each of the four blocks of variables are briefly described in the following sections and the Supplementary Materials. The overall study design and analytic workflow are summarized in Fig. 1.

### Clinical assessment

All participants underwent structured clinical interviews conducted by trained psychologists using the Diagnostic Interview for Genetic Studies (DIGS). ED was assessed via the Affective Lability Scale (ALS) and the Cognitive Emotion Regulation Questionnaire (CERQ). Affective lability, also referred to as emotional instability, represents a core maladaptive ED pattern characterized by rapid, frequent, and intense fluctuations in emotional states [67]. Cognitive emotion regulation strategies were specifically evaluated through the CERQ by calculating the ratio of non-adaptive strategies to total emotion regulation strategies (adaptive plus non-adaptive), following established approaches in the literature [68]. Hence, in all analyses, ED was operationalized by two continuous indicators: (i) the ALS total score and (ii) the CERQ non-adaptive ratio (non-adaptive subscales divided by the total CERQ score), with higher values on both reflecting greater ED. Additional clinical measures included depression, anxiety, personality, and other clinical, cognitive, and demographic questionnaires (Supplementary Table 1).

### Biological markers

Serum markers were quantified using the Procarta multiplex immunoassay platform. These included, for instance, proinflammatory (e.g. C-reactive protein, CRP), anti-inflammatory (e.g. interleukin-10, IL-10), and neurotrophic markers (e.g. Brain Derived Neurotrophic Factor, BDNF). Details of sample collection, assay procedures, and quality control are provided in the Supplementary Materials.

### Dynamic functional connectivity markers: acquisition and micro-coactivation patterns (μCAPs) computation

Detailed methods for resting-state fMRI data acquisition, preprocessing, seed-based μCAPs analysis for dynamic functional connectivity (dFC) of hippocampal subregions, and motion correction procedures are provided in the Supplementary Materials and our prior publications [37, 56]. Briefly, five large-scale networks (i.e. μCAPs), each connected with a specific data-driven, activity-based hippocampal parcel were identified through μCAPs dFC analysis. Occurrences of each network were computed per each participant, indicating the dFC of that μCAP with the associated hippocampal parcel (Supplementary Figure 2).

### Structural connectivity markers: acquisition and fractional anisotropy (FA) computation

Diffusion-weighted images were acquired using a 3 T Siemens TrioTim scanner (TR = 6300 ms, TE = 84 ms, voxel size = 2.3 × 2.3 × 2 mm, 72 diffusion directions, b = 1000 s/mm², 52 slices, FoV read = 230 mm, phase = 100%). Whole-brain FA values were extracted using the ENIGMA-DTI pipeline [69]. This included preprocessing with FSL’s tract-based spatial statistics (TBSS) and registration to the ENIGMA-DTI FA template, followed by ROI-wise extraction using the JHU (Johns Hopkins University) white matter atlas [70]. For bilateral ROIs, left and right FA values were averaged to obtain a single bilateral estimate per tract, in order to reduce dimensionality and increase robustness to noise. Further details on the preprocessing and TBSS analysis are provided in the Supplementary Materials.

### Descriptive analysis: PCA

PCA was conducted in two steps: first, PCA was performed on the full dataset, including all clinical (CL), hippocampal dynamic functional connectivity (dFC), structural connectivity (white matter integrity, FA), and serum biomarkers (BIO) variables, to identify integrated components capturing shared variance across modalities. Retained components (RCs) scores were compared across transdiagnostic groups (EDD, EDDoff, and HC) using ANOVAs followed by Tukey’s HSD post-hoc tests. This analysis aimed to investigate multimodal and transdiagnostic patterns of EDD vulnerability. Because the EDD group included individuals with BD, BPD, or ADHD, diagnosis-specific analyses were conducted (see Supplementary Methods and Results). Second, to explore domain-specific contributions and associations, separate PCAs were conducted on each of the four aforementioned blocks (CL, dFC, FA, and BIO). FDR-adjusted Pearson correlations were then computed between RCs from different blocks to investigate cross-domain associations.

This two-step approach enabled both the identification of overarching multimodal patterns, and the interpretation of inter-block, domain-specific correlations among retained components from different blocks.

For each PCA, the number of components to retain was determined using the parallel analysis criterion [71, 72], which modifies Kaiser’s criterion to adjust for small-sample bias in eigenvalue sizes, and performs a permutation test to evaluate the importance of eigenvalues against the 95th percentile of random eigenvalues. The analyses were carried out in R (version 4.3.3) using the ‘psych‘ package, with Varimax rotation and Thurstone’s method applied to compute regression-based component loadings for each block.

### Predictive analysis: exploratory random forest classification of group and symptoms prediction

The machine learning RF approach was employed to firstly predict group, and secondly ED symptoms (detailed in the Supplementary Materials). Briefly, in the first analysis, all multimodal predictors (clinical, biological, and neuroimaging markers) were used to train a RF model using the randomForest package in R for discriminating HC from individuals with either EDD or familial risk for EDD (EDDoff). Models were trained on a stratified 70/30 train–test split, using the out-of-bag procedure for internal validation, with standard RF settings (e.g. mtry = √p) and performance summarized by accuracy, balanced accuracy, Cohen’s κ, and AUC on the held-out test set.

The RF model performance was benchmarked against a baseline linear model, implemented using ridge regression, with additional analyses performed to ensure robustness against overfitting (see Supplementary Materials and Supplementary Results).

In the second analysis, two RF regressions were trained to predict continuous ED symptom scores (ALS and CERQ) from non-clinical features (serum and neuroimaging markers only).

Further methodological details for all the aforementioned analyses, including procedures to address potential confounding factors and to minimize overfitting in the RF models, are provided in the Supplementary Materials. Briefly, potential confounders were assessed by comparing groups on demographic and clinical variables, testing associations between framewise displacement and hippocampal μCAP occurrence to evaluate motion effects, examining the contribution of age to PCA components and RF feature-importance rankings, and correlating psychotropic medication load with retained PCA component scores showing significant group differences.

## Results

### Demographics and clinical characteristics

The final sample consisted of 237 participants: 97 individuals diagnosed with EDD (including 30 with ADHD, 33 with BPD, and 34 with BD), 67 offspring of different EDD patients (EDDoff), and 73 HC. The groups did not differ significantly in terms of educational attainment or handedness (all *p* > 0.05). A summary of key demographic and clinical characteristics for each group is presented in Table 1, as well as in our previous publications [56, 66]. Further details on the sample, subgroups, confounding analyses and a list of detailed abbreviations are in the Supplementary Results. Briefly, sex distribution, handedness, BMI, and cognitive scores did not differ significantly across groups; motion was not associated with hippocampal μCAP occurrence; age was not among the most influential RF predictors; and psychotropic medication load was not significantly associated with PCA components showing group differences.

**Table 1:** Participants’ demographic and clinical characteristics.

	EDD patients (N = 97)	EDD offspring (N = 67)	Controls (N = 73)
***Demographics***			
Age: mean (SD)	27.4 (8.4)^a^	20.3 (3.3)^ab^	24.9 (8.6)^b^
% females	62.9	55.2	56.2
% left-handed	6.2	9	12.3
BMI: mean (SD)	21.8 (5.1)	21.4 (3.7)	22.4 (3.9)
***Main Clinical scores***			
ALS: mean (SD)	1.4 (0.62)^ab^	0.9 (0.6)^ac^	0.5 (0.3)^bc^
CERQ (ratio non-adaptive/total): mean (SD)	0.41 (0.09)^ab^	0.38 (0.08)^a^	0.35 (0.07)^b^
Reverse digit span: mean (SD)	8.1 (1.6)	8.1 (2)	8.4 (2.1)

The table summarizes main demographic and clinical characteristics of EDD patients, EDD offspring, and healthy controls. Values sharing the same letter as superscripts (i.e. “a” or “b” or “c”) are significantly different from each other (*p* < 0.05, ANOVA or chi-square tests).

*ALS*, affective lability scale; *BMI*, body mass index; *CERQ*, cognitive emotion regulation questionnaire, *EDD*, emotion dysregulation disorders; *SD*, standard deviation.

### Significant group differences in PCA components related to ED symptoms, inflammation, and hippocampal connectivity

PCA performed on the full multimodal dataset (clinical, neuroimaging, and serum markers) yielded eight retained significant components (RCs). Of these, two RCs, detailed in Supplementary Figure 3, showed significant group differences and are the main focus of this section.

**Fig. 2:**
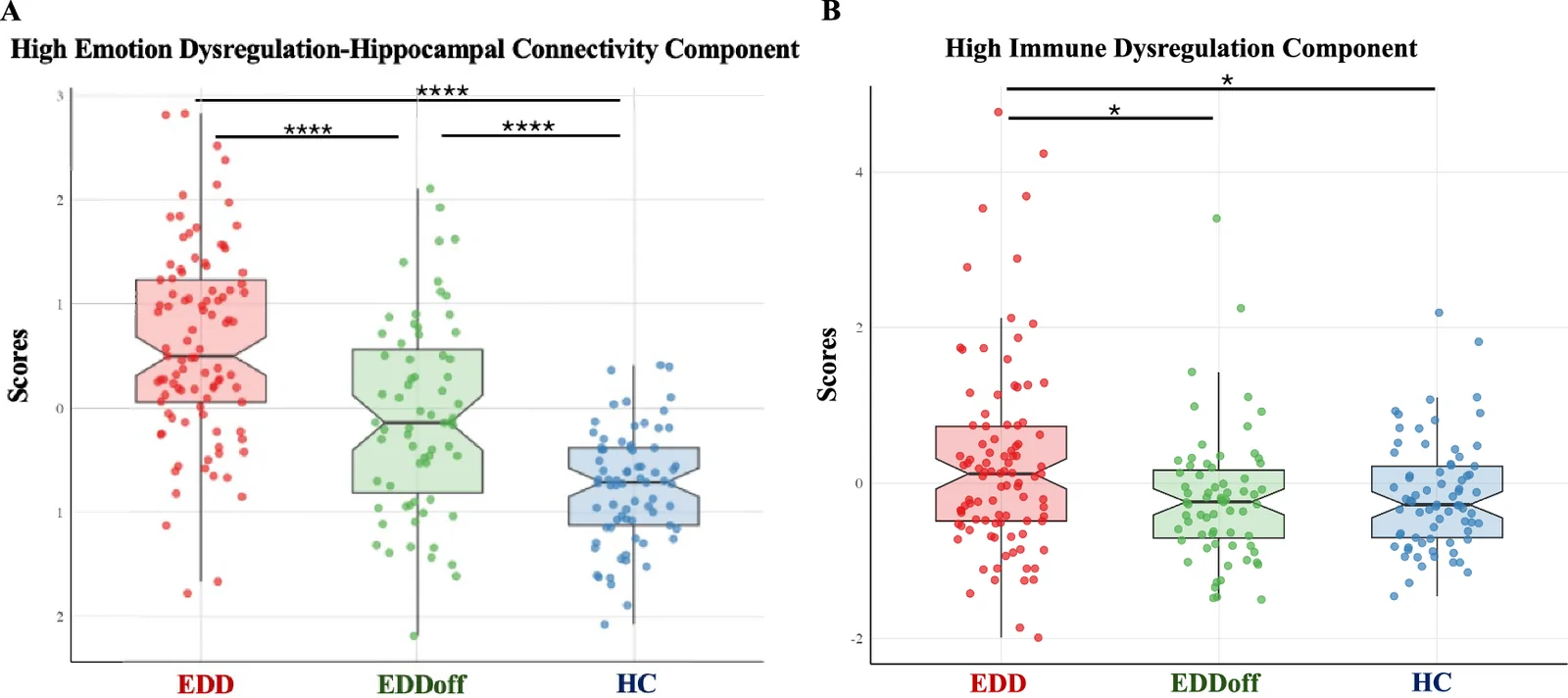
Group differences in multimodal components related to emotion dysregulation and immune dysregulation. (**A**) Notched boxplots illustrating group differences in the multimodal “High emotion dysregulation–hippocampal dFC” component (RC3) derived from PCA on the full dataset. RC3 scores are shown for EDD patients (red), offspring of EDD patients (EDDoff, green), and healthy controls (HC, blue). A robust main effect of group was observed (F(2,234) = 58.29, p < 0.001). Tukey-adjusted post-hoc tests indicated a clear gradient, with EDD patients scoring higher than both EDDoff and HC (*p*_*adj*_ = 8.2 × 10⁻^7^ and *p*_*adj*_ < 1 × 10⁻^15^, respectively), and EDDoff also scoring higher than HC (*p*_*adj*_ = 5.7 × 10⁻^6^). This component was characterized by increased affective lability, anxiety, borderline traits, and non-adaptive emotion regulation strategies, accompanied by reduced hippocampal connectivity with the visual–sensorimotor network (SMN) and increased connectivity with the limbic network (LN). Significant pairwise differences are indicated by asterisks (****, *p*_*adj*_ < 0.0001). **(B)** Boxplots illustrating significant group differences in the multimodal “High immune dysregulation” component (RC5) from PCA on the full dataset. Notched boxplots display RC5 scores across the same three groups (EDD, EDDoff, HC; colors as in A). Scores were significantly elevated in EDD patients relative to both EDDoff and HC (*p*_*adj*_ < 0.02 for all Tukey-adjusted post-hoc comparisons). This component was predominantly driven by pro-inflammatory markers (e.g., MIF, MCP1, CRP, IL18, IL17) and the neurotrophic factor BDNF. Significant group differences are denoted by asterisks (*, p_*adj*_ < *0.05)*.

The third component (RC3), characterized by high affective lability, non-adaptive emotion regulation strategies, anxiety, and borderline personality traits, along with reduced hippocampal connectivity with the SMN and increased connectivity with the LN, was labeled “Elevated ED–SMN Hypoconnectivity–LN Hyperconnectivity.” This multimodal component (RC3) showed a robust main effect of group (F(2,234) = 58.29, p < 0.001). Tukey-adjusted post-hoc tests indicated a clear gradient, with EDD patients scoring higher than both EDDoff and HC (*p*_*adj*_ = 8.2 × 10⁻^7^ and *p*_*adj*_ < 1 × 10⁻^15^, respectively), and EDDoff also scoring higher than HC (*p*_*adj*_ = 5.7 × 10⁻^6^; Fig. 2A). Thus, this component discriminates all three groups, reflecting a graded increase in multimodal emotion-dysregulation–related burden from HC to EDDoff to EDD.

The fifth component (RC5), labeled “High Immune Dysregulation,” was predominantly driven by pro-inflammatory immune markers (e.g., MIF, MCP1, CD62E, CRP, IL18, IL17) and included the neurotrophic factor BDNF, as well as some mediators that can have both pro- and anti-inflammatory roles, like IL2-R and EGF. Scores for this component were significantly higher in EDD patients compared to both EDDoff and HC (*p*_*adj*_ < 0.02 for all comparisons, Fig. 2B).

Collectively, these multimodal findings demonstrate distinct clinical, functional connectivity, and biological alterations characterizing EDD patients and EDDoff relative to healthy individuals.

### Inflammation is inversely correlated with white matter integrity, and hippocampal connectivity positively correlates with ED symptoms and inflammation

To explore the interrelations between PCA-derived components between blocks of variables, we computed PCA separately within each block of variables, revealing a total of 11 significant RCs: four components from biological serum markers, three from FA-based structural connectivity, three from clinical variables, and one from hippocampal dFC.

Pearson correlations between retained components from the block-level PCAs revealed significant inter-block relationships.

**Fig. 3:**
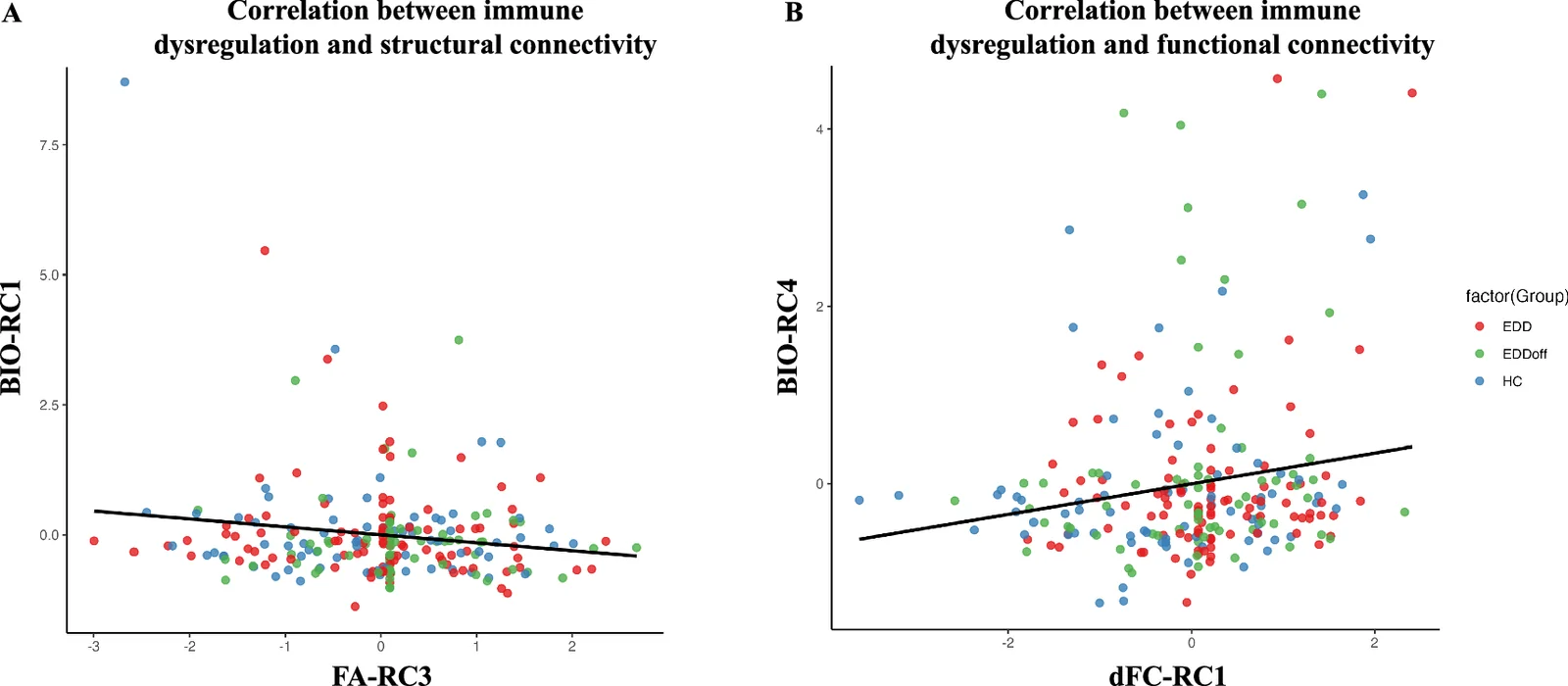
Scatterplots illustrating correlations between neuroimaging markers (structural and functional connectivity) and immune dysregulation. (**A**) Scatterplot showing the significant negative correlation between white matter integrity and immune dysregulation. Higher immune dysregulation (BIO-RC1, driven by immune markers IL12, IL4, IFNg, IL10, IL17, IL1b, and TNFa) transdiagnostically correlated with reduced white matter integrity (fractional anisotropy, FA-RC3, driven by tracts such as CGH, SS, EC, RLIC, PTR), r(235) = − 0.15, *p*_*adj*_ = 0.01. **(B**) Scatterplot showing the significant positive correlation between hippocampal dynamic functional connectivity (dFC) and systemic inflammation. Scores on the hippocampal dFC component (dFC-RC1) increase with rising scores on the pro-inflammatory biological component (BIO-RC4), driven by IP-10, GROα, VCAM, IL-18, and IL-17, r(235) = 0.17, *p*_*adj*_ = 0.0076.

A negative correlation emerged between a structural connectivity component (FA-RC3, featuring high FA in CGH, SS, EC, RLIC, PTR, Supplementary Figure 5) and an immune dysregulation component (BIO-RC1, mainly driven by immune markers, such as IL12, IL4, IFNg, IL10, IL17, IL1b, and TNFa, Supplementary Figure 6), *r* = −0.15, *t* = −2.38, *p*_*adj*_ = 0.01, Fig. 3A. However, a sensitivity analysis removing one influential HC subject reduced the strength of this correlation to a non-significant negative trend.

**Fig. 4:**
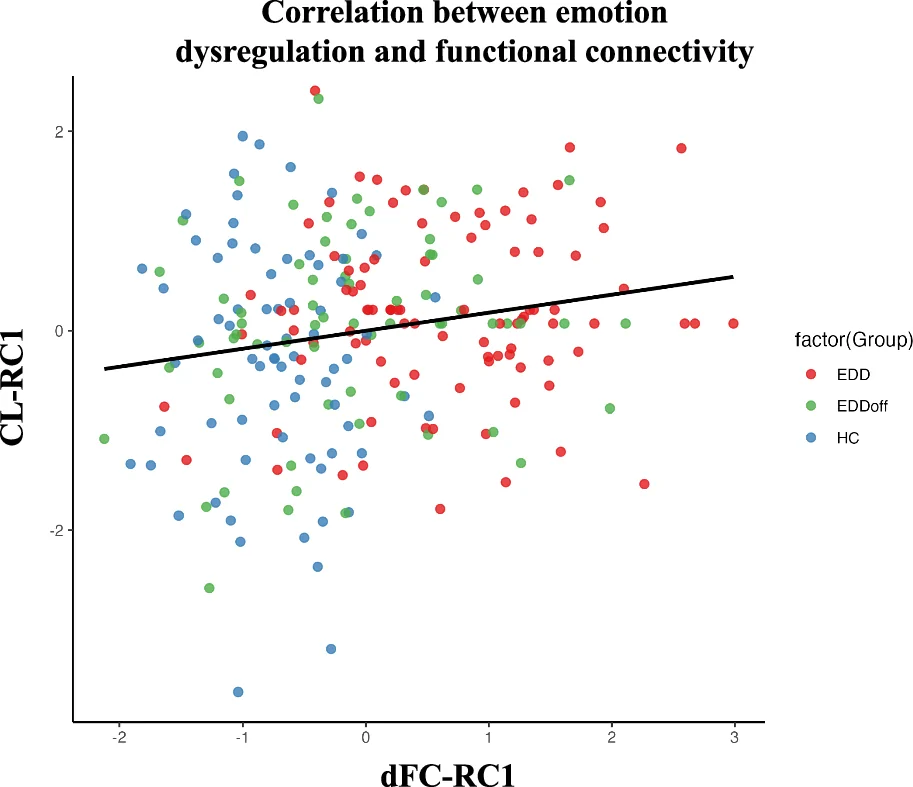
Scatterplot showing the significant positive correlation between clinical emotion dysregulation (CL-RC1) and hippocampal dynamic functional connectivity (dFC-RC1). Higher clinical symptoms of emotion dysregulation (CL-RC1, mainly driven by affective lability, anxiety, borderline personality traits) correlated with increased disruptions in hippocampal dFC, r(235) = 0.18, *p*_*adj*_ = 0.0051.

There was only one dFC component (dFC-RC1) that was positively correlated with a clinical “Elevated ED” component (CL-RC1, Supplementary Figure 7) and with an inflammatory component (BIO-RC4, driven by pro-inflammatory immune markers, such as IP10, GROa, VCAM, IL18, IL17, Supplementary Figure 8), *p*_*adj*_ < 0.01 for all comparisons (Fig. 3B and Fig. 4).

Detailed variable loadings and description for all retained components from the unimodal (clinical, FA, dFC, and biological) and multimodal PCAs are provided in the Supplementary Results and in the Supplementary Figures 16–29.

### An exploratory RF classifier distinguishes HC with good accuracy

A RF classifier was trained to distinguish HC from the individuals with either EDD or familial EDD risk (EDDoff), using all multimodal features (listed in Supplementary Table 1). The model achieved a high classification balanced accuracy of 80.3%, accuracy of 85.7% (95% CI [75.3%, 92.9%], *p* = 0.001), with substantial agreement (Cohen’s κ = 0.64) and non-significant misclassification bias (McNemar’s p = 0.34). Discriminative performance was also fair, with an AUC of 0.88 (95% CI [0.81, 0.96]). Sensitivity was 93.9%, specificity 66.7%. Positive predictive value was 86.8%, and negative predictive value was 82.4%. The full confusion matrix is reported in Supplementary Figure 9.

Feature importance analysis (Supplementary Figure 10) indicated that the most influential variables in predicting group membership were clinical symptom measures related to ED (e.g., affective lability, borderline personality traits, depression, anxiety, and ADHD symptoms), followed by neuroimaging-derived features including structural (e.g. FA of the RLIC and of the ACR) and functional connectivity measures (such as SMN and visual connectivity). CRP, IL2R, and MCP-1 were the most influential serum marker contributors to the model.

### Exploratory RF regression accurately predicts ED symptoms

A RF regression model was trained to predict ED-specific clinical scores, in particular individual scores on the ALS, using only biological and neuroimaging markers as input features. The model demonstrated reliable performance, yielding an RMSE of 0.54, MAE of 0.44, and R² = 0.23. Predicted ALS scores were highly significantly correlated with observed scores (*r* = 0.48, *t* = 4.45, *p*_*adj*_ < 0.00005, Supplementary Figure 11), indicating transdiagnostic convergence between model outputs and actual affective lability severity in the whole sample.

In addition, predicted scores on the CERQ, derived from the same set of biological and imaging predictors, were also significantly correlated with actual values (*r* = 0.32, *t* = 2.73, *p*_*adj*_ = 0.007, Supplementary Figure 12), further supporting the predictive capacity of these multimodal markers for ED symptoms and cognitive emotion regulation strategies. In contrast, the same feature set failed to accurately predict other clinical symptom domains (e.g. depression or ADHD symptoms).

**Fig. 5:**
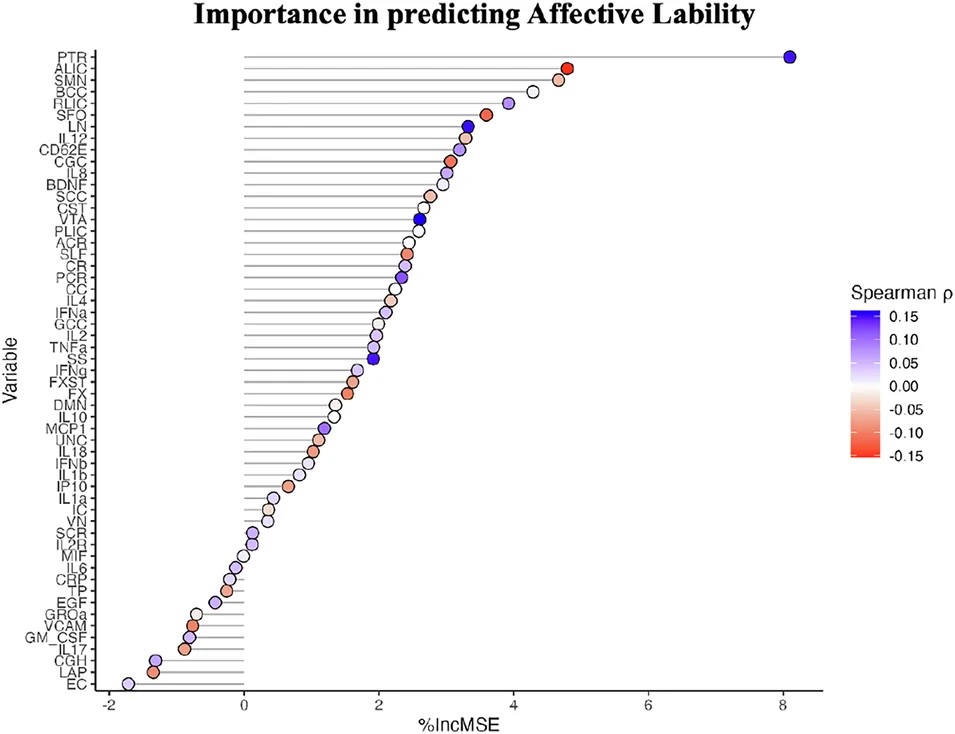
Feature importance for predicting affective lability (ALS) from neuroimaging and biological features. Variables are ranked by importance (measured as percentage of increase in Mean Squared Error with permutation, %IncMSE) from the random forest regression model predicting Affective Lability Scale (ALS) scores. Higher %IncMSE values indicate greater predictive contribution. Top predictors included posterior thalamic radiation (PTR) fractional anisotropy (FA), anterior limb of the internal capsule FA (ALIC), sensorimotor network connectivity (SMN), body of the corpus callosum FA (BCC), and retrolenticular internal capsule FA (RLIC). Color coding reflects Spearman correlation (ρ) between each feature and ALS scores, blue for positive and red for negative associations. Please refer to Supplementary Table 1 for the complete list of all variables and corresponding abbreviations.

Feature importance rankings highlighted neuroimaging features as the most influential in the prediction of ED symptoms (prediction models for ALS in Fig. 5, for CERQ in Supplementary Figure 13, respectively). In particular, high FA in the PTR and low anterior limb of the internal capsule (ALIC) FA were the most important predictors of affective lability, while low SMN connectivity and high LN connectivity with the hippocampus were the most important functional connectivity predictors of affective lability. Detailed feature importance rankings for CERQ prediction model are presented in Supplementary Figure 13, but structural connectivity measures were the most influential predictors also in this case.

## Discussion

This multimodal, transdiagnostic study identified clinically and biologically meaningful signatures of ED vulnerability and disease across EDD patients, EDDoff, and healthy controls.

As detailed above, PCA conducted on the full dataset identified two transdiagnostic and multimodal components (one associated with ED and hippocampal connectivity, and the other associated with inflammation) that showed significant differences among groups. Subsequent modality-specific PCAs and inter-block correlation analyses revealed clinically relevant patterns of convergence across clinical, neuroimaging, and biological domains. Complementary machine learning analyses further confirmed the predictive utility of specific multimodal features for both group classification and symptom severity, extending prior literature and supporting a dimensional, transdiagnostic view of ED vulnerability.

### Multimodal PCA points to ED symptoms and hippocampal connectivity as markers of vulnerability to EDD

Clinically, EDD patients and EDDoff exhibited elevated scores in the “Elevated ED–SMN Hypoconnectivity–LN Hyperconnectivity” component (RC3) characterized by affective lability, anxiety, borderline traits, and non-adaptive emotion regulation strategies, along with reduced hippocampal connectivity with the visual-SMN and increased connectivity with the LN (Fig. 2a).

Since all pairwise group differences were strongly significant, the distinct, intermediate phenotype observed in EDDoff supports the hypothesis that disruptions in hippocampal connectivity and ED symptoms may reflect a transdiagnostic vulnerability marker, present even in the absence of full-blown clinical syndromes. This pattern could represent a common liability that does not necessarily lead to disease expression but may increase susceptibility when combined with additional risk factors [56]. In fact, this is not only consistent with the conceptualization of ED as a transdiagnostic feature with multiple components across psychiatric disorders and vulnerable individuals [1], but also confirms that opposite patterns of hippocampal connectivity alterations in the SMN and LN may represent an EDD fMRI vulnerability marker. Indeed, also in previous literature [46–55, 57, 73] and in our own prior work on dFC in a subset of this population [56], disruptions in the SMN and LN connectivity were associated with EDD and EDD vulnerability, both transdiagnostically and in BD patients and their offspring. In particular, in BD patients only we confirmed the pattern of disrupted SMN connectivity also using traditional CAPs analysis, which considers the hippocampal seed as homogenous. Thus, while the hippocampal dFC features build on our previous work, all other data and findings reported here, including the multimodal components, structural and biological findings, cross-domain correlations, and machine-learning analyses, are entirely novel.

### Multimodal PCA points to immune dysregulation as a marker of full-blown EDD

**Fig. 6:**
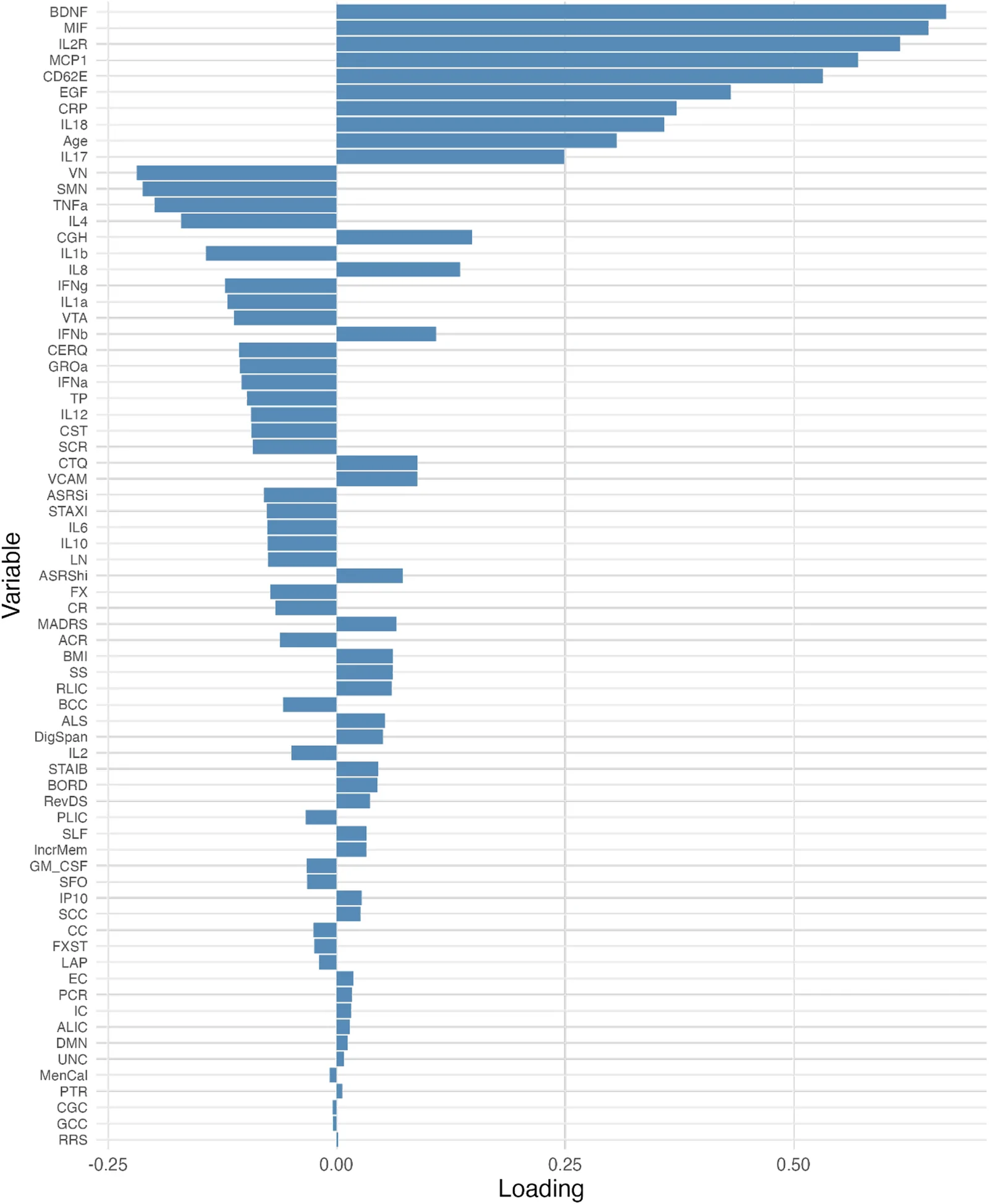
Variable loadings on the “High Immune Dysregulation” fifth retained principal component (RC5) from the PCA performed on the full multimodal dataset. Horizontal bars show each variable’s standardized loading on RC5, sorted by absolute magnitude. Bars extending to the right indicate positive loadings, driven primarily by immune and neurotrophic markers (e.g. BDNF, IL2R, MIF, MCP1, CD62E, EGF, CRP, IL18, IL17). Please refer to Supplementary Table 1 for the complete list of all variables and corresponding abbreviations.

Biologically, a distinct “High immune dysregulation” profile was increased in EDD patients compared to the other two groups (Fig. 2B). This component (RC5, Fig. 6) was primarily driven by classical pro-inflammatory markers (e.g., MIF, MCP1, CD62E, CRP, IL18, IL17) and also by the neurotrophic factor BDNF. This heightened inflammatory profile aligns with established literature on systemic immune dysregulation in EDD [26, 27, 51, 74]. In particular, several markers driving RC5, such as peripheral CRP, MCP-1, and IL2R, have been consistently found to be elevated in BD, ADHD or BPD patients [26, 27, 51, 74].

Interestingly, a minority of the drivers of this multimodal component (RC5) included markers that may also have an anti-inflammatory role (e.g., IL2R and EGF). This indicates that chronic immune dysregulation, characterized by simultaneous activation of both inflammatory and reactive immunomodulatory responses over extended periods, rather than transient purely pro-inflammatory states, may be associated with EDD. This interpretation aligns with evidence highlighting persistent immune imbalance in individuals with EDD, linked for instance with chronic stress [26, 27, 51, 74].

Elevated peripheral BDNF in EDD patients could reflect prior exposure to psychotropic medications, such as mood stabilizers, antidepressants, or antipsychotics, which are known to upregulate BDNF expression [75–77]. Alternatively, increased BDNF may reflect a compensatory neurotrophic response to inflammation or stress-induced neuronal damage, consistent with BDNF’s dual neuroprotective and anti-inflammatory roles [78, 79].

The elevated inflammatory profile characterized by this component indicates that immune dysregulation may mark established EDD, rather than simple EDD vulnerability, which was captured instead by the aforementioned “Elevated ED–SMN Hypoconnectivity–LN Hyperconnectivity” component.

### Modality-specific PCA reveals that hippocampal connectivity is positively correlated with ED symptoms and inflammation, while immune dysregulation may be inversely associated with white matter integrity

Modality-specific PCA allowed for the exploration of inter-block correlations, revealing positive correlations between hippocampal dFC and both clinical ED symptoms and pro-inflammatory immune markers (BIO-RC4, mainly driven by IP10, GROa, VCAM, IL18, IL17). These findings further implicate hippocampal dFC, more than, for instance, structural connectivity, in ED symptoms. This is consistent with models positing a bidirectional cycle wherein ED symptoms promote stress and inflammation (e.g. via HPA axis dysregulation), which in turn disrupt emotion-regulating neural networks, thereby exacerbating ED and perpetuating the vicious cycle [27, 74]. Although this interpretation remains speculative and no causality can be inferred from the present study, exploratory evidence from Mendelian Randomization studies has causally implicated inflammation in the development of EDD, such as BD [80, 81].

Supporting this model, a component driven by high immune dysregulation (BIO-RC1, characterized by IL12, IL4, IFNg, IL10, IL17, IL1b, and TNFa) tended to be negatively associated with a white matter FA component (FA-RC3), driven by FA in tracts such as the CGH first of all, but also the SS, EC, RLIC, and PTR (Fig. 3A). The CGH is a cortico-limbic bundle connecting the parahippocampal gyrus with the cingulate cortex. It plays a crucial role in integrating cognitive, emotional, and memory-related processes. Disruption in the CGH may impair top-down regulation of emotional responses, especially the modulation of limbic activity by the prefrontal cortex [82]. It has been implicated in EDD and is often associated with difficulties in emotional control and regulation [82]. The other white matter tracts (SS, EC, RLIC, PTR) are involved in visuospatial processing and sensory-motor integration, whose disruption may contribute to impaired processing of emotionally salient stimuli, integration of perceptual and emotional information, exacerbating ED.

Although the association between immune dysregulation and white matter integrity should be interpreted cautiously, as the finding is not robust enough to support strong mechanistic conclusions in the present sample, this reinforces the hypothesis that immune dysregulation may contribute to microstructural white matter disruption in emotion regulation circuits. Selective vulnerability of emotion regulation tracts, particularly the CGH, to peripheral inflammation has been previously proposed [63, 64, 83, 84]. Possible mechanisms include differences in metabolic demand, oligodendrocyte density, and delayed myelination, as these tracts are among the last to complete development [85].

Interestingly, the immune component BIO-RC1 negatively associated with FA was not limited to pro-inflammatory markers but included a minority of anti-inflammatory cytokines (e.g., IL-4, IL-10), suggesting that white matter degradation may result from prolonged immune dysregulation, triggering both inflammatory and immunomodulatory responses over the span of years, rather than acute inflammation alone. This aligns with the aforementioned evidence suggesting that chronic dysregulation of immune signaling, rather than transient inflammatory states, plays a key role in the pathophysiology of EDD and related structural brain changes [79].

Overall, these findings highlight converging disruptions across immune, structural, and functional systems that may underpin transdiagnostic vulnerability to ED and support the continued investigation of multimodal biomarkers in high-risk populations such as EDDoff.

### Machine learning analysis accurately predicted group status

Machine learning models further validated the clinical significance of these findings. The RF classifier achieved strong accuracy in distinguishing HC from individuals with either EDD or familial EDD risk (EDDoff). While prior studies have focused on predicting specific diagnoses, for instance, showing that multimodal machine learning models can predict ADHD diagnosis with varying accuracies [86], our study is, to our knowledge, the first to apply a transdiagnostic model that successfully predicts participant status based on multimodal features.

While exploratory and not yet suitable for clinical screening (despite the model’s high sensitivity), the analysis offers two key insights. First, it highlights the discriminative power of multimodal data in identifying EDD risk or diagnosis, supporting dimensional and transdiagnostic frameworks. Second, it enables the identification of the most influential predictors of classification, providing insight into EDD pathophysiology. As expected, clinical measures of ED were among the top contributors. Notably, white matter microstructure (particularly FA in the RLIC and ACR) and pro-inflammatory markers (e.g., CRP and MCP-1) also emerged as key features. As mentioned, peripheral CRP and MCP-1 have indeed been consistently found to be elevated in EDD patients [26, 51, 74] and were crucial components in the multimodal PCA described above. Concerning FA markers, RLIC carries fibers from the thalamus (which is strictly connected with the limbic system) to the parietal and occipital lobes, including optic radiations and projections relevant for sensory processing [87]. By mediating visual and sensory input to higher-order cortical areas, the RLIC is involved in integrating perceptual and emotional information [88]. Alterations could lead to heightened sensitivity to environmental stimuli, a hallmark of emotional lability. Notably, the RLIC was also a central driver of the FA component inversely correlated with systemic inflammation, as discussed earlier.

The ACR, on the other hand, connects the anterior cingulate cortex and prefrontal cortex, key regions for emotion regulation, with subcortical limbic structures such as the thalamus and striatum. This connectivity supports top-down modulation of emotional responses, and reduced structural connectivity (FA) in the ACR has been observed in EDD, such as BD and BPD [89, 90], suggesting that impaired ACR white matter integrity may underlie ED. Notably, interventions aimed at enhancing emotion regulation, such as mindfulness-based practices, have been shown to improve structural connectivity (FA) in the ACR [91].

Collectively, these findings converge with the PCA results, underscoring the significance of white matter tracts, particularly the RLIC and ACR, in EDD, the potential vulnerability of RLIC structural connectivity to inflammation, and their promise as transdiagnostic targets for research and interventions in individuals with or at risk for EDD.

### Machine learning models accurately predicted ED symptoms

RF regression analyses showed that multimodal biological and neuroimaging markers specifically predicted individual differences in ED, measured as affective lability (also called emotional lability, a core component of ED [67]) and cognitive emotion regulation strategies, but not other psychopathological dimensions (e.g., depression, ADHD symptoms), highlighting specificity to ED vulnerability.

Feature importance analyses revealed that lower FA in the ALIC, reduced visual-SMN hippocampal connectivity, higher FA in the PTR, and increased LN hippocampal connectivity were particularly influential in predicting higher affective lability.

The ALIC is a critical white matter bundle connecting prefrontal cortical areas to subcortical structures, including parts of the limbic system [87]. It supports executive and cognitive control functions essential for effective emotion regulation and adaptive behavioral responses [92]. Lower FA in the ALIC, indicative of reduced white matter integrity and disrupted fronto-subcortical connectivity, may therefore compromise the ability of the prefrontal cortex to modulate automatic emotional responses from the limbic system, leading to LN hyperactivity and to more rapid and less controlled emotional shifts, characteristic of affective lability.

The SMN described in our analysis encompassed parietal and occipital regions, involved in processing and integrating multimodal sensory inputs essential for coherent perceptual experiences and visual-sensorimotor integration [93]. Impaired integration of visual-sensorimotor information with contextual memory (e.g. in the case of disrupted SMN-hippocampus connectivity) may thus hinder the ability to appropriately appraise emotional situations in light of past emotional experiences, impairing accurate sensory integration and modulation of emotional reactivity and contributing directly to emotional dysregulation [46–55, 57, 73], as we previously discussed [56]. Interestingly, as discussed above, also one of the most important predictors of ALS, i.e. RLIC, is involved in sensory processing, underscoring its importance in ED symptoms pathophysiology.

Similarly to the RLIC, also the PTR, a major white matter pathway, connects thalamic nuclei to some of the parietal and occipital regions involved in the SMN. Functionally, the PTR supports the relay of visual and somatosensory information from subcortical structures to cortical processing regions involved in sensory integration [94]. While decreased FA typically indicates impaired structural integrity, higher FA in the PTR observed in our study may reflect atypical or maladaptive hyper-organization or maturational trajectories [95]. We hypothesize that this hyperconnectivity could lead to heightened and less filtered sensory input leading to emotional reactivity to the environment, overwhelming the normal processing capacities of the SMN. Consequently, such maladaptive reorganization might impair efficient sensorimotor integration and sensory gating, increasing emotional reactivity and instability.

Hence, in this unified mechanistic model, higher FA in the PTR may speculatively be associated with compensatory maladaptive disruption of SMN connectivity, ultimately manifesting as increased affective lability and ED, reflected in the higher LN connectivity without a corresponding modulatory activation of the frontal lobes, given the white matter impairment in the ALIC. The convergence of these structural and functional disruptions within the sensorimotor-emotional integration circuits underscores the network-based nature of ED vulnerability, providing an integrated target for future research and early interventions addressing ED symptoms.

### Strengths and limitations

The present study has several notable strengths. First, the integration of multimodal data, including clinical, neuroimaging, and biological measures, allows for a comprehensive characterization of ED vulnerability across multiple domains. Second, the inclusion of at-risk offspring provides unique insight into potential intermediate phenotypes, which is valuable for identifying early markers of psychopathology risk. Third, employing advanced machine learning approaches such as RF models significantly enhances the ability to detect complex nonlinear interactions among multimodal data. Finally, the transdiagnostic approach enables the identification of targets suitable for addressing the shared vulnerability among offspring of patients with a certain EDD. Nevertheless, certain limitations should be acknowledged. Restricting μCAPs analyses to a hippocampal seed may have led us to miss broader, brain-wide dFC alterations that seed-free approaches might have revealed. Nonetheless, we note that hippocampal μCAPs still provide system-level information by characterizing how distinct hippocampal subregions dynamically interact with large-scale networks, rather than examining the hippocampus in isolation from the rest of the brain. The cross-sectional design precludes definitive causal inference about the progression from vulnerability to clinical manifestation; longitudinal studies are thus needed to confirm and extend these findings. Given the multimodal nature of the dataset and the modest sample size relative to the number of included variables, bootstrap-based estimates of loading stability were not performed, and the PCA analyses are primarily descriptive. Because RC3 includes clinical ED measures, group differences on this component should not be interpreted as evidence for a purely non-clinical biomarker. Rather, RC3 captures a clinical–functional connectivity pattern in which ED symptoms co-vary with hippocampal network alterations. Non-clinical biomarker evidence is addressed separately through the modality-specific PCA results, inter-block correlations, and RF regression models predicting ED symptoms from imaging and serum markers only. The RF classification and regression models were developed and evaluated within a single cohort, without external validation in an independent sample. Although we used a stratified train–test split, out-of-bag error estimates, and dummy noise variables to investigate potential overfitting, these internal procedures cannot fully eliminate optimism bias in performance metrics or ensure generalizability to other populations. While the sample size is adequate for exploratory machine learning analyses, replication in larger and independent cohorts will be essential to strengthen the robustness and external validity of these findings. For these reasons, the ML results should be regarded as exploratory and hypothesis-generating, rather than as definitive predictive models ready for clinical application. Moreover, before any such multimodal, resource-intensive risk assessment could be considered for clinical use, its potential benefits would need to be carefully weighed against costs, participant burden, and ethical risks (including stigma, anxiety, or inappropriate labeling of asymptomatic offspring), with clear safeguards on how probabilistic risk information is communicated and used in care. Although distinguishing EDDoff from EDD patients using non-clinical markers would be highly relevant for identifying brain or biological patterns associated with transition from familial risk to manifest disorder, the present sample size does not allow such an analysis to be performed reliably. Future longitudinal studies with larger at-risk cohorts will be needed to determine which neural, immune, or multimodal patterns predict conversion from familial vulnerability to clinically expressed EDD. Finally, although our subanalyses did not reveal significant influence of potential confounding variables (e.g., age, sex, BMI, cognitive scores, handedness, medication, or participant movement during scanning; detailed in the Supplementary Materials), unmeasured confounding variables (e.g., environmental stressors, sleep quality, past medication effects, nutrition etc.) might influence the observed multimodal signatures, warranting consideration in future research.

## Conclusions

These findings collectively suggest that hippocampal connectivity alterations and clinical symptoms may represent ED vulnerability markers, as evidenced by their disruption in both EDD patients and EDDoff compared to HC, along with their positive intercorrelation, which suggests their mutual reinforcement in early-stage vulnerability. In contrast, a pronounced pro-inflammatory state appears to emerge specifically in established EDD, suggesting that significant immune dysregulation may be more characteristic of full clinical manifestation rather than vulnerability stages.

Several multimodal features emerge as potential transdiagnostic EDD markers to be further investigated for early intervention, risk stratification, patient stratification, or diagnosis. These include white matter microstructure in visual/sensorimotor (PTR, RLIC), and emotion-regulating (ALIC, CGH) tracts, functional connectivity disruptions in large-scale networks (particularly visual-sensorimotor, SMN, and limbic, LN, networks), and elevated levels of systemic biomarkers including BDNF and pro-inflammatory markers (CRP, MCP-1, MIF). The transdiagnostic convergence of ED, visual-sensorimotor and emotion-regulating networks dysfunction, and immune dysregulation provides novel mechanistic insights into the multi-system pathophysiology underlying EDD.

This integrated vulnerability profile carries implications for developing potential precision psychiatry approaches aimed at early identification and intervention in high-risk populations [22]. Future longitudinal studies should systematically evaluate the predictive value and causal relationships of these multimodal markers for early identification and illness progression, while intervention research should investigate targeted approaches capable of modifying these transdiagnostic risk pathways. As a perspective, particularly promising in this regard may be mindfulness-based interventions, given their potential capacity to improve functional connectivity patterns [96, 97], reduce inflammatory markers [98], and enhance white matter integrity [91, 96, 99]. Hypothetically, these effects may reflect a fundamental restoration of body-mind integration [100], potentially addressing the observed sensorimotor dysconnectivity and its downstream clinical effects on emotional processing and dissociation symptoms in EDD patients. Hence, a multimodal, transdiagnostic, and targeted intervention of this nature could theoretically interrupt the vicious cycle sustaining ED.

This integrated approach could inform future psychiatric strategies, moving beyond diagnosis to the early identification and targeted, early treatment of at-risk individuals, ultimately reducing vulnerability and progression to overt psychopathology among at-risk populations such as EDDoff.

## Supplementary information

supporting information

## Data Availability

The data that support the findings of this study are available from the corresponding author upon reasonable request.
