# Supplementary material for: Machine learning reveals a multimodal, transdiagnostic signature of emotion dysregulation vulnerability across patients, offspring, and controls
Source: Mol Psychiatry. 2026 Jul 27;31(12):7144–56. doi: 10.1038/s41380-026-03767-1 (PMC13736645; doi:10.1038/s41380-026-03767-1)
Supplement: Supplementary file 1 [file 41380_2026_3767_MOESM1_ESM.docx]

**Machine Learning Reveals a Multimodal, Transdiagnostic Signature of Emotion Dysregulation Vulnerability Across Patients, Offspring, and Controls**

**Running head:** Multimodal Markers of Emotion Dysregulation

**List of authors**

Luigi F Saccaro^a,b,e*^; Thomas Larrieu^c^; Farnaz Delavari^d,e^; Céline Pellaton^g^; Ben Meuleman^a,h^; Nader Perroud^a,b^; Dimitri Van De Ville^e,f^; Nicolas Toni^c^; Camille Piguet^a,i^

**Affiliations**

^a^ Psychiatry Department, Faculty of Medicine, University of Geneva, Switzerland

^b^ Psychiatry Department, Geneva University Hospital, Switzerland

^c^ Center for Psychiatric Neuroscience, Department of Psychiatry, Lausanne University Hospital, University of Lausanne, Prilly, Switzerland

^d^ Developmental Imaging and Psychopathology Laboratory, University of Geneva School of Medicine, Geneva, Switzerland

^e^ Medical Image Processing Laboratory, Neuro-X Institute, École Polytechnique Fédérale de Lausanne, Geneva, Switzerland

^f^ Department of Radiology and Medical Informatics, Faculty of Medicine, University of Geneva, Geneva, Switzerland

^g^ Division of Immunology and Allergy, Lausanne University Hospital (CHUV) and University of Lausanne, Lausanne, Switzerland.

^h^ Swiss Center for Affective Sciences, University of Geneva, Geneva, Switzerland

^i^ General Pediatric Division, Geneva University Hospital, Switzerland

***Corresponding author:** Luigi Francesco Saccaro, MD, Psychiatry Department, Faculty of Medicine, University of Geneva, Switzerland; Psychiatry Division, Geneva University Hospital, Rue Gabrielle-Perret-Gentil 4, 1205 Geneva, Switzerland; email: [LuigiFrancesco.Saccaro@unige.ch](mailto:LuigiFrancesco.Saccaro@unige.ch)

**Contents**

[SUPPLEMENTARY BACKGROUND	5](#_Toc229229952)

[Shared features call for a transdiagnostic study of emotion dysregulation in BD, BPD, and ADHD	5](#_Toc229229953)

[Rationale for Focusing on Hippocampal Dynamic Functional Connectivity and whole-brain structural connectivity	6](#_Toc229229954)

[SUPPLEMENTARY METHODS	9](#_Toc229229955)

[Participants’ clinical evaluation and exclusion criteria	9](#_Toc229229956)

[Serum markers: multiplex bead-based immunoassay	10](#_Toc229229957)

[Resting-state fMRI acquisition and analysis of hippocampal dynamic functional connectivity with micro-CoActivation Patterns (μCAPs)	11](#_Toc229229958)

[MRI acquisition	12](#_Toc229229959)

[Preprocessing of functional MRI data	12](#_Toc229229960)

[fMRI seed selection	13](#_Toc229229961)

[Micro-CAPs (μCAPs) analysis	13](#_Toc229229962)

[Diffusion MRI analysis: structural connectivity (fractional anisotropy)	17](#_Toc229229963)

[Statistical data preprocessing and general statistical details	19](#_Toc229229964)

[Further details on RF model for group prediction	22](#_Toc229229965)

[Baseline group predictor model: ridge regression	22](#_Toc229229966)

[Further details on RF model for clinical symptoms prediction	23](#_Toc229229967)

[Code availability	23](#_Toc229229968)

[Figures and graphs	24](#_Toc229229969)

[SUPPLEMENTARY RESULTS AND DISCUSSION	25](#_Toc229229970)

[Details on the subgroups	25](#_Toc229229971)

[Identification of the micro-coactivation patterns (μCAPs) and the corresponding hippocampal subdivisions	25](#_Toc229229972)

[Potential confounders	26](#_Toc229229973)

[Baseline linear model (ridge regression) for group prediction	28](#_Toc229229974)

[Negative control features confirm RF models validity	28](#_Toc229229975)

[RF prediction of cognitive emotion regulation strategies	29](#_Toc229229976)

[Group differences in the within-block PCA	29](#_Toc229229977)

[Multimodal diagnostic subgroup differences	30](#_Toc229229978)

[Correlations among the retained components of multimodal PCA	31](#_Toc229229979)

[Further details on retained components of within-block PCAs	31](#_Toc229229980)

[Further details on retained components of the multimodal PCA	34](#_Toc229229981)

[SUPPLEMENTARY TABLES AND SUPPLEMENTARY TABLES LEGENDS	37](#_Toc229229982)

[SUPPLEMENTARY FIGURES AND SUPPLEMENTARY FIGURES LEGENDS	42](#_Toc229229983)

[SUPPLEMENTARY REFERENCES	77](#_Toc229229984)

# SUPPLEMENTARY BACKGROUND

## Shared features call for a transdiagnostic study of emotion dysregulation in BD, BPD, and ADHD

Identifying transdiagnostic, multimodal markers of vulnerability to emotion dysregulation (ED) specifically in bipolar disorder (BD), borderline personality disorder (BPD), and attention-deficit/hyperactivity disorder (ADHD), collectively referred to here as emotion dysregulation disorders (EDD), holds significant clinical relevance for several reasons, as previously described(1). Firstly, these disorders are notably prevalent and associated with considerable impairment in daily functioning and quality of life(2,3). Secondly, EDD exhibit a meaningful degree of genetic heritability(4–8), suggesting shared underlying biological mechanisms. Thirdly, existing literature underscores substantial overlap in symptoms and psychopathological profiles across EDD, which frequently co-occur. For instance, offspring of individuals diagnosed with BD may exhibit increased risk not only for BD but also for BPD and ADHD(9–17). Lastly, since EDD typically emerge during youth and emotion-regulating circuits mature until young adulthood(18,19), EDD represent ideal targets for early intervention strategies targeting ED(20,21). The urgency of transdiagnostic early markers to reduce the burden of these disorders is further underscored by the high prevalence(3,22) of these conditions, ranging from 2.5–15% for ADHD(23), 0.8–2.4% for BD(24), and 2.7–6% for BPD(25), and their profound individual and societal burden. Although early interventions have demonstrated promise(21), identifying robust early markers has proven challenging due to the complex, multifactorial nature of EDD risk.

Overall, a growing body of research supports the conceptual and clinical overlap between bipolar disorder (BD), borderline personality disorder (BPD), and attention-deficit/hyperactivity disorder (ADHD), particularly with respect to emotion dysregulation (ED) as a shared core feature. Eich et al. (2014) demonstrated comparable levels of cyclothymic temperament (a marker of affective lability) in all three conditions, reinforcing affective instability as a common transdiagnostic trait(26). Neuroimaging findings by Kebets et al. (2021) further substantiated this convergence, revealing a shared fronto-limbic neural signature of ED across BD, BPD, and ADHD, particularly in regions implicated in emotion regulation and impulse control(27). The ongoing DynAMoND study aims to deepen this understanding through fine-grained digital phenotyping and genetic analyses of affective dysregulation dynamics across the three disorders(28). Importantly, shared therapeutic avenues have also emerged: Durpoix et al. (2023) reported that a transdiagnostic dialectical behavior therapy (DBT)-informed group intervention significantly improved emotion regulation in individuals with BD, BPD, or ADHD, with sustained effects at one-year follow-up(29). Collectively, these studies highlight common pathophysiological mechanisms and treatment targets, reinforcing the clinical value of a unified, transdiagnostic approach to ED in these populations.

## Rationale for Focusing on Hippocampal Dynamic Functional Connectivity and whole-brain structural connectivity

While, as described below and in the main text, this study adopted a whole-brain approach to assess white matter microstructural alterations through fractional anisotropy (FA), dynamic functional connectivity (dFC) analyses were intentionally focused on the hippocampus. This decision was motivated by extensive theoretical and empirical evidence positioning the hippocampus as a key hub in emotion regulation (ER) networks(30,31). The hippocampus integrates affective, cognitive, and sensorimotor information via its anterior–posterior axis, and it is central to fronto-limbic circuits regulating emotion and stress(32–34). It is also uniquely sensitive to peripheral influences such as inflammatory signaling and stress hormones, two domains implicated in the pathophysiology of emotion dysregulation disorders (EDD)(35–39,39–41).

Given this convergence of functional, structural, and immunological relevance(42–50), the hippocampus is a strong candidate region for investigating shared neural vulnerability mechanisms across BD, BPD, or ADHD.

While prior studies have explored hippocampal connectivity in BD(51–53), transdiagnostic and developmental extensions (particularly involving at-risk offspring) remain scarce. Importantly, as noted in the manuscript, we have previously identified altered hippocampal co-activation dynamics using micro-co-activation pattern analysis (μCAP, described below) in individuals with EDD(1). These prior findings demonstrated that disruptions in hippocampal μCAPs are associated with emotion dysregulation symptoms and may reflect a mechanistically meaningful vulnerability marker across disorders, which we further investigate in this paper from a multimodal standpoint. The μCAPs method provides a refined, data-driven characterization of hippocampal functional states by decomposing its spontaneous co-activation into distinct spatial and temporal patterns. Compared to static or sliding-window connectivity approaches, μCAPs offer higher sensitivity to transient neural events and regional heterogeneity, particularly relevant for the hippocampus, whose subregions are linked to different regulatory functions and symptom domains.

In contrast, the structural domain was interrogated using a whole-brain FA framework, which allows for unbiased identification of widespread white matter alterations potentially affecting multiple networks relevant to ED. This choice is particularly justified by the hypothesis that immune dysregulation (implicated in EDD pathophysiology, as described above) may exert diffuse neurotoxic effects on myelinated pathways throughout the brain, rather than targeting specific tracts. Whole-brain FA analysis therefore provides a comprehensive lens to capture such global microstructural vulnerability. Together, this multimodal strategy enables high-resolution investigation of both focal (hippocampal) and distributed (white matter) neural correlates of transdiagnostic ED vulnerability.

# SUPPLEMENTARY METHODS

## Participants’ clinical evaluation and exclusion criteria

The main exclusion criteria comprised: 1) presence of a neurological disorder; 2) inability to provide informed consent; 3) current delirium or dementia; 4) other severe medical conditions; and 5) contraindications to undergoing MRI scanning. Although no strict age or gender criteria were imposed, participants were primarily aged between 15 and 65, reflecting the typical patient demographic attending the Mood and Emotion Dysregulation Clinics within the Psychiatry Department at Geneva University Hospitals. Participants with intellectual disabilities were excluded using the Diagnostic Interview for Genetic Studies (DIGS), which includes assessments for intellectual functioning, attention, and comorbidities(54). Additionally, individuals presenting psychotic disorders other than BD were excluded. Patients diagnosed with BD were required to have stable pharmacological treatments and maintain remission status for at least four weeks prior to the study, defined by scores lower than 6 on the Young Mania Rating Scale (YMRS) and lower than 13 on the Montgomery-Åsberg Depression Rating Scale (MADRS).

Before the scanning session, participants underwent comprehensive clinical and cognitive assessments. Clinical symptom measures included: the Montgomery–Åsberg Depression Rating Scale (MADRS) for depressive symptoms; the Trait Anger Expression Inventory (STAXI) for anger; the Borderline Symptom Checklist (BORD) for borderline personality features; the Cognitive Emotion Regulation Questionnaire (CERQ) non-adaptive to total strategies ratio(55,56); the Childhood Trauma Questionnaire (CTQ) for early adversity; the Ruminative Response Scale (RRS) for rumination; the Trait Anxiety Inventory (STAI-B) for trait anxiety; the Adult ADHD Self-Report Scales for inattentive (ASRSi) and hyperactive (ASRShi) symptoms; the Temperament Evaluation of Memphis, Pisa, Paris, and San Diego Scale (TEMPS) for affective temperament traits; and the Affective Lability Scale (ALS) for affective instability(57).

Cognitive performance was assessed using standardized working memory and executive function tasks: the Digit Span (DigSpan) and Reverse Digit Span (RevDS) tests, an Incremental Memory Task (IncrMem) requiring participants to reorder a sequence of digits from smallest to largest, and a Mental Calculation (MenCal) task measuring mental arithmetic abilities.

Chronological age in years, sex, and body mass index (BMI) were also acquired for each participant. All participants identified as cisgender. All participants provided written informed consent under the ethical approval CER 13-081 granted by Geneva University Ethical Committee.

## Serum markers: multiplex bead-based immunoassay

Biological samples (serum) were collected before the scanning session from all participants and stored at −80°C until analysis. All procedures were conducted in accordance with institutional and international ethical guidelines (ethic committee approval number: CER 13–081). To quantify multiple inflammatory markers simultaneously, we used a multiplex bead-based immunoassay from Thermo Fisher Scientific (Human ProcartaPlex Mix&Match, PPX-23). The panel included 28 neurotrophic and inflammatory proteins: Brain-Derived Neurotrophic Factor (BDNF); adhesion molecules such as E-selectin (CD62E) and VCAM-1; acute phase proteins like C-reactive protein (CRP); cytokines and chemokines including IL-1α, IL-1β, IL-2, IL-2R, IL-4, IL-6, IL-8, IL-10, IL-12, IL-13, IL-1RA, IL-17a, IL-18, TNF-α, IFN-α, IFN-β, and IFN-γ; growth factors (EGF, GM-CSF); and additional inflammatory mediators (GROα, IP10, LAP, MCP1, MIF). Please refer to **Supplementary Table 1** for the complete list of all variable names and corresponding abbreviations used in the manuscript. Assays were performed according to the manufacturer's instructions. Briefly, standards and samples randomized across plates by two co-authors (CP and TL) who were fully blinded to diagnostic groups, were incubated with antibody-coated magnetic beads in a 96-well plate. Following incubation and washing steps, biotinylated detection antibody mix was added, and, after incubation and washing, streptavidin-PE was added. The final signal was acquired using a Luminex Bio-Plex200 (BioRad Laboratories Inc.) instrument. Raw fluorescence data were acquired using Bio-Plex Manager software (Biorad Laboratories Inc.). Standard curves were generated based on the known concentrations provided in the assay kit, and sample concentrations were computed using a five-parameter logistic (5-PL) regression model. Analyte values falling below the lower limit of detection were assigned a value of zero. Two biomarkers (IL-13 and IL-1RA) were excluded from further analyses due to a high proportion (i.e., >50%) of undetectable or missing values. This resulted in a final set of 26 immune and neurotrophic markers included in the analysis (see **Supplementary Table 1** for the complete list). All samples were run in duplicate, and intra- and inter-assay coefficients of variation were monitored to ensure data quality.

## Resting-state fMRI acquisition and analysis of hippocampal dynamic functional connectivity with micro-CoActivation Patterns (μCAPs)

Given the well-recognized role of the hippocampus in emotion regulation and EDD in general(51–53,58), detailed in the Introduction, we focused on hippocampal functional connectivity. Hippocampal dynamic functional connectivity (dFC) was quantified using micro-coactivation patterns analysis (μCAPs). The μCAPs method described in the following sections partly overlap with our previous publications employing similar methodology(51,52), but we provide complete details below for the sake of clarity.

### MRI acquisition

Whole-brain MRI data were acquired immediately following completion of the clinical questionnaires, with a 3T scanner (Siemens TIM Trio), and a 32 channels head-coil, at the Brain & Behavior Laboratory (BBL), University of Geneva. The Blood Oxygenation Level Dependent (BOLD) contrast was measured using a T2*-weighted echo-planar sequence (EPI). 250 functional volumes of 36 axial slices each (TR/TE/flip angle = 2100ms/30ms/80°, FOV=205 mm, resolution=64×64, isotropic voxels of 3.2 mm3, distance factor 20%) were acquired. Furthermore, we collected a high-resolution T1-weighted anatomical image [TR/TI/TE/flip angle=1900ms/900ms/2.27ms/9°, FOV=256mm, resolution=256×256, slice thickness=1mm3, 192 sagittal slices, phase encoding direction= Anterior-Posterior (AP)]. Our sequence included dummy scans (~ 9s) at the beginning of the fMRI scanning. The subjects were instructed to lie awake, with normal breathing, and not think about anything in particular for about 8 minutes.

### Preprocessing of functional MRI data

MATLAB 2018a (MathWorks, Inc., Natick, MA, USA) was used to preprocess structural and functional images using SPM12 (Wellcome Trust Centre for Neuroimaging, London, UK; http://www.fil.ion.ucl.ac.uk/spm) in accordance with a previously described workflow(59).

Specifically, functional images were realigned, followed by spatial smoothing using a 3 mm full width at half maximum isotropic Gaussian kernel. The structural scans were co-registered to the mean functional image. The structural images were segmented using SPM12's segmentation algorithm(60), and Diffeomorphic Anatomical Registration using Exponentiated Lie algebra (DARTEL)(61) was used to create a study-specific template. The first 5 functional scans were excluded to allow for magnetization equilibration. To remove confounding effects, linear detrending was applied to the time series, and the motion parameters (translation and rotation) along with global, mean white matter, and mean cerebrospinal fluid signals were regressed out using the warped DPARSF tissue masks(62). The time series were then bandpass filtered to 0.01-0.10 Hz. After preprocessing, all functional images were warped to the DARTEL space and subsequently to Montreal Neurological Institute space for population-based analysis. Additionally, the µCAPs method included extended motion correction by identifying frames with high framewise displacement (FD > 0.5 mm(63)) and excluding them from the analysis.

### fMRI seed selection

For the initial seed selection, we employed a comprehensive parcellation of the bilateral hippocampus, supported by anatomical data from the Harvard Oxford atlas and functional connectivity data from both task-related and resting-state studies. This was validated through meta-analytic connectivity mapping using two distinct databases, neurosynth and brainMap(33,64).

### Micro-CAPs (μCAPs) analysis

We then conducted μCAPs analysis, which identifies the contributions of different subsections of the seed region to specific patterns of synchronous brain activity, known as co-activation patterns (CAPs). Unlike CAPs analysis, which treats the seed region as uniform(65), μCAPs analysis uses a data-driven approach to detect variations within the seed for each candidate brain pattern. This iterative process identifies a certain number (K) of μCAPs, each linked to a weighted seed-map, allowing various parts of the seed to contribute differently to the pattern of co-activation. The μCAPs analysis code is accessible at: <https://github.com/MIPLabCH/mCAP> and is comprehensively detailed elsewhere(66), as well as in the following sections below.

Briefly, this approach enables a more precise characterization of the hippocampal functional units, identifying the specific contributions of its subdivisions to each dynamic brain pattern (μCAPs). Using a data-driven "test-retest" procedure, we identified five μCAPs strongly co-activating with five hippocampal subdivisions (**Supplementary Table 1**). This procedure, further described below, determined that K=5 was the optimal number of distinct brain clusters in terms of replicability for this dataset. Frames assigned to every μCAP were traced back to the participant’s time course, and the occurrences of every μCAP within subjects were calculated and extracted for the following analyses. In this context, occurrences indicate the cumulative time points (frames) associated with a specific μCAP across the whole time course. Essentially, occurrences indicate how frequently a subject's brain activity matched a particular spatial configuration represented by a certain μCAP, while the corresponding seed hippocampal subregion was also active.

a) *Co-activation patterns (CAPs) analysis*

The µCAPs method builds on the well-established CAPs technique, which we will briefly recapitulate here. As previously discussed(52,65,67), through a spatiotemporal framewise functional magnetic resonance imaging (fMRI) deconvolution, CAPs analysis disentangles distinct functional brain networks interacting with the seed area. This approach conceptually aligns with the model of discrete transitions between brain meta-states(68) and its physiological soundness is supported by multiple works, as previously described(67). In contrast to stationary connectivity studies that disregard time-varying data, clustering neural activity variations into spatiotemporally overlapping patterns of brain activation is captured by CAPs analysis. These patterns of brain activation are therefore not considered mutually exclusive. Additionally, in contrast to other techniques for analyzing dynamic functional connectivity (dFC), CAPs concentrate on individual fMRI volumes rather than fMRI time courses. As a result, they are extremely sensitive in recognizing and examining transient, recurrent patterns of co-activation and their temporal variability(37,56)(65,67).

Previous studies(65,69) have provided thorough descriptions of CAPs analysis. In summary, CAPs analysis chooses the time points with "higher than threshold" activity by extracting and Z-scoring the average seed BOLD signal throughout the fMRI time series. The retained whole-brain volumes from each subject are then agnostically classified using a K-means clustering algorithm into discrete clusters that represent the CAPs; within-cluster differences, which are defined as spatial similarity, were found to be smaller than those across clusters (further information regarding clustering can be found elsewhere(70) and in the sections that follow). The magnitude of their divergence from zero is then measured by converting these CAPs into spatial Z-maps.

b) *Initialization and seed focalization*

As described in detail elsewhere(52,71), μCAPs analyze BOLD fMRI data employing a matrix that represents the time-dependent fMRI volumes for all subjects, in voxels. A subset of the voxels is defined as the seed, or the region of interest, in this case the bilateral hippocampus. The goal is to identify a number (“*K”)* of micro co-activation patterns (µCAPs) associated with weight maps for the seed. At the first step, the arithmetic mean of BOLD signal within all voxels of the initial seed is calculated and used as the first time course for frame-selection. At following iterative steps, the time course for each seed weight map is computed by averaging the fMRI volumes weighted by the corresponding seed weights, which are created by a winner-takes-all approach across all K µCAPs (see section *e) Seed weight maps update* below). Therefore, allowing smaller seed regions to have an equally great role in frame-selection in the next steps.

*c) Criteria for selection of frames*

In this iterative process, a threshold for the Z-scored time course for each seed weight map is defined, and if activity exceeds this threshold, the corresponding fMRI volumes are tagged as "selected." The initial threshold is set to 0.5 for maximum sensitivity, and in subsequent iterations, it is set to 1.0, a typical value used in conventional CAP analyses(65,72).

*d) Clustering*

A K-means method is used to cluster all fMRI volumes that were chosen based on the seed activation using a 1-correlation distance measure. A micro co-activation pattern (µCAP) is represented by the centroid of every K-cluster. In this instance, the K-means is performed for a candidate number of clusters ranging from K = 2: 20. The optimal number of clusters (K) is determined through a test-retest algorithm including a 20-fold random 50-50 split of subjects into training and testing subsamples described in detail elsewhere(71). Briefly, both the training and test frames undergo K-means clustering to generate K distinct centroids. The K clusters from the test and training frames are then matched using the Hungarian algorithm, and the average distance between matched centroids is calculated for a range of K values (from 1 to 20). The optimal K is determined by identifying the K value that yields the lowest average distance between matched centroids, indicating maximal replicability between the clustering results of the test and training frames.

*e) Seed weight maps update*

To update the seed weight maps for the next iteration, each µCAP is restricted to the seed region and its values are assigned to the corresponding seed weights. We adopt a "winner-takes-all" approach by keeping only the largest µCAP value for each voxel and setting values for other µCAPs to zero. The algorithm then iterates, going to frame-selection again, until convergence is achieved.

*f) Convergence*

Convergence was defined as the point for which the distance between the seed patterns of matching μCAPs of the previous and the new step was less than 0.005. The distance is computed using the Hungarian algorithm(73) and averaged cosine distance across iterations. The first iteration (in which the seed pattern is uniform), is not included in the convergence assessment.

g) *Motion correction*

Two steps were included in our technique to address any motion artifacts. Firstly, we excluded one participant with poor image quality or considerable translational or rotational motion. Additionally, we used a scrubbing procedure that adheres to recognized guidelines(63) to ensure that the patterns of μCAPs are not obtained from frames that are interrupted by movement. To lessen the effect of motion-related distortions, frames having a FD exceeding 0.5—which could be determined using the Power formula(63) —were eliminated. The percentage of scrubbed frames were in any case lower of 5% for each participant.

## Diffusion MRI analysis: structural connectivity (fractional anisotropy)

As mentioned in the main text, fractional anisotropy (FA) values were extracted using the ENIGMA-DTI pipeline (available at https://enigma.ini.usc.edu/protocols/dti-protocols/). In detail, T1-weighted images first underwent N4 bias-field correction using ANTs, followed by skull stripping with FreeSurfer to obtain a brain-extracted T1 image. Diffusion-weighted images were then preprocessed using FSL and MRtrix3. A b0 image was extracted and brain extraction was performed using BET to derive a b0-based brain mask, and eddy-current–related distortions and subject motion were corrected with FSL’s *eddy_openmp*, using subject-specific acqp and index files and the original bvec/bval files, with appropriate rotation of b-vectors. After eddy correction, the masked b0 image was upsampled to 1 mm isotropic resolution and used to estimate linear registration (9 degrees of freedom, FLIRT) to the T1 brain.

To further improve diffusion-to-T1 alignment, we performed nonlinear registration of the diffusion data to the T1 brain using ANTs (SyN) and applied the resulting deformation fields to the entire corrected DWI time series. (antsApplyTransforms). In this distortion-corrected and T1-aligned diffusion space, diffusion tensors were estimated using MRtrix3’s dwi2tensor, and FA, mean diffusivity, axial diffusivity, and radial diffusivity maps were computed using tensor2metric. Quality control was performed visually at each step (raw DWI, post-eddy correction, registrations, tensor and FA maps). The resulting FA maps were then entered into FSL’s tract-based spatial statistics (TBSS(74)), which included nonlinear registration to the ENIGMA-DTI FA template, skeletonization, and projection of individual FA maps onto the common white matter skeleton. ROI-wise FA values were extracted from the skeleton using the JHU (Johns Hopkins University) white matter atlas, as adapted in the ENIGMA-DTI protocol, for the major tracts: Anterior Corona Radiata (ACR), Anterior Limb of Internal Capsule (ALIC), Body of the Corpus Callosum (BCC), Corpus Callosum (CC), Cingulum bundle (CGC), Perihippocampal Cingulum (CGH), Corona Radiata (CR), Corticospinal Tract (CST), External Capsule (EC), Fornix (FX), Fornix Stria Terminalis (FXST), Genu of the Corpus Callosum (GCC), Internal Capsule (IC), Posterior Corona Radiata (PCR), Posterior Limb of the Internal Capsule (PLIC), Posterior Thalamic Radiation (PTR), Retrolenticular part of Internal Capsule (RLIC), Splenium of the Corpus Callosum (SCC), Superior Corona Radiata (SCR), Superior Fronto-Occipital Fasciculus (SFO), Superior Longitudinal Fasciculus (SLF), Sagittal Stratum (SS, including parts of the inferior fronto-occipital fasciculus), Tapetum (TP), and Uncinate Fasciculus (UNC). Please see **Supplementary Table 1** for the complete list in alphabetical order and to **Supplementary Figure 30** for depiction of the main ROIs highlighted in the results. For bilateral ROIs, left and right FA values were averaged to yield a single bilateral estimate per tract, in order to reduce dimensionality and increase robustness to noise.

## Statistical data preprocessing and general statistical details

All statistical analyses and data processing were carried out in R version 4.3.3 (2024-02-29) using the following main packages: *readxl* and *openxlsx* for data import/export; *psych* and *paran* for principal‐components and parallel‐analysis; *dplyr* and *tidyr* for data manipulation; *ggplot2*, *grid*, and *ggpubr* for plotting; *multcomp* for post‐hoc Tukey tests; *caret* for model training and resampling; *randomForest* for classification and regression; and *stringr* for string formatting. All features were Z-scored before PCA and random forest (RF) analyses. To address missing data, we applied group-average imputation within each diagnostic group in R. This method preserves group-level variance and is generally recommended when group membership is expected to be a meaningful predictor of variable distributions and there is up to about 20% missing data(75), as is the case in this study where missingness was low, similarly distributed among groups, less than 12% in clinical and biological variables, 15% in dFC variables, and 18% in FA variables. We employed Spearman correlations for all correlations between variables which showed a significant departure from normality based on the Shapiro-Wilk test and visual inspection of QQ-plots, and Pearson correlations in other cases.

We investigated groups differences with one-way ANOVAs and post-hoc Tukey test adjustment or chi-square tests where appropriate.

In particular, we also investigated subgroup differences further across the eight multimodal retained components (RC1–RC8) using one-way analyses of variance (ANOVAs), with diagnostic group (CTRL, BD, BDoff, BPD, BPDoff, ADHD, ADHDoff) as the between-subjects factor. Homogeneity of variance was assessed with Levene's test prior to each ANOVA. For each component yielding a significant omnibus effect, pairwise post-hoc comparisons were conducted using Tukey's honestly significant difference (HSD) procedure to control the family-wise error rate. Unadjusted pairwise p-values were additionally corrected using the Benjamini-Hochberg false discovery rate (FDR) procedure. These analyses were performed in R using the `emmeans` and `rstatix` packages. The significance threshold was set at p < .05.

RFs were used for prediction in two steps, first for categorical group prediction (classifier) and second for continuous clinical symptoms prediction (regression). We chose RF as our primary machine learner for several reasons, namely **(a)** that it is flexible in adapting to nonlinear patterns, **(b)** that it is well-suited to high-dimensional predictor sets, using a stepwise algorithm that targets only the most predictive variables, **(c)** that it is relatively resistant to overfitting, due to its out-of-bag (OOB) optimization, and **(d)** that it requires relatively little tuning in its control parameters (76).

In both RF models, described in further detail below, the full dataset was randomly partitioned into a training set (70%) and a held-out test set (30%), using stratified sampling on the group factor to preserve class proportions. No observations from the test set were used at any point during model fitting, hyperparameter choice, or out-of-bag (OOB) error estimation. Within the training set, RF employed the standard bootstrap-based OOB procedure: each tree was trained on a bootstrap resample of the training data, and OOB predictions for each observation were obtained by aggregating only those trees that had not seen that observation in their bootstrap sample. This OOB estimate provided an internal cross-validated assessment of training error and guided the choice of a sufficiently large number of trees (ntree, increased until the OOB error curve plateaued, in agreement with existing literature(76–79)), but it was never used for formal model selection or tuning. To limit overfitting and because our primary aim was exploratory pattern discovery and variable importance mapping, we did not perform other cross-validation for hyperparameter optimization. Instead, we followed standard recommendations and existing literature(76–79) and set the number of candidate predictors considered at each split to the rule-of-thumb value mtry = √p (where p is the number of predictors), which corresponds to the default for classification in the original randomForest implementation and has been widely used in biomedical RF applications(80). All available predictors (excluding identifiers such as ID and group labels) were entered into the RF models; we did not apply any external supervised feature selection prior to model fitting. Thus, feature selection was implicit and embedded in the RF algorithm through random sub-sampling of predictors at each split, and variable importance indices (e.g. %IncMSE) were computed post hoc to interpret which clinical, imaging, or immune variables contributed most to the predictions, without feeding back into model construction. Model performance was evaluated only on the held-out test set, using overall accuracy, 95% confidence intervals for accuracy, sensitivity, specificity, balanced accuracy, Cohen’s κ, and the area under the ROC curve (AUC) for the classifier, and standard regression metrics (RMSE, R²) for the regression models. To formally test whether the classifier performed better than chance, we used the no-information-rate test provided by caret::confusionMatrix, which assesses whether the observed accuracy is significantly higher than the accuracy expected from always predicting the majority class. Given this design and the absence of external validation, the RF results should be interpreted as exploratory and hypothesis-generating, providing convergent information on potentially relevant multimodal markers of EDD vulnerability rather than as fully optimized predictive tools.

## Further details on RF model for group prediction

In the first RF analysis, multimodal predictors (clinical, biological, and neuroimaging) were used to train a RF model for group classification (healthy controls (HC) vs the other two groups). Data were stratified by group and split into training (70%) and test (30%) sets. Three artificial "probe" variables (Gaussian, uniform, and binomial noise) were initially included to detect overfitting. Any model that ranked these probes among its top predictors would be considered unreliable.

This RF model was trained using the *randomForest* package in R with 1000 trees, based on stabilization of the OOB classification error, in agreement with existing literature(76–79). The number of drawn candidate variables in each split was set to the standard value of sqrt(p)(81). Variable importance was computed as %IncMSE and mean decrease in accuracy, i.e., the relative contribution of each input variable to classification performance. The RF model performance was compared with a baseline linear model, implemented with ridge regression, described in the following section.

## Baseline group predictor model: ridge regression

To benchmark the performance of the RF classifier against a baseline linear model, we implemented a ridge regression to distinguish HC from the individuals with either EDD or familial EDD risk (EDDoff). Ridge regression consists of a traditional logistic regression with an additional L2-regularization penalty. This penalty constrains the overall size of model coefficients and allows for stable predictions even when the predictor set is high-dimensional and possibly collinear. Prior to model fitting, all numeric features were standardized using z-score normalization to ensure uniform scaling and prevent bias due to differing feature magnitudes. Ridge regression was performed using the glmnet package in R. Model tuning was conducted via 10-fold cross-validation within the training set to identify the optimal regularization parameter. Both the ridge and RF models were trained and evaluated using the same stratified 70/30 training-to-test split, enabling direct and fair comparison of classification performance.

## Further details on RF model for clinical symptoms prediction

For the second RF analysis, two separate RF regression models were constructed to predict continuous ED symptom scores, specifically ALS and the CERQ (ratio of the non-adaptive emotion regulation strategies score to total). The predictor variables for these models consisted solely of non-clinical features, including serum and neuroimaging markers. Each RF regressor was trained using 500 trees, based on stabilization of the OOB classification error for all models, and the number of randomly selected predictors at each split was set to a third of the total number of predictor variables, a common heuristic for regression tasks in the randomForest package(81). The predictive accuracy of each model was evaluated using Root-Mean Squared Error (RMSE), Mean Absolute Error (MAE), coefficient of determination (R^2^), and Pearson's correlation coefficient between the predicted and observed symptom scores. Permutation-based variable importance, quantified as the percentage increase in mean squared error (%IncMSE) derived from the OOB samples, was also extracted to assess the relative contribution of each predictor.

## Code availability

As mentioned in the main text, fractional anisotropy (FA) values were extracted using the ENIGMA-DTI pipeline, which is available at https://enigma.ini.usc.edu/protocols/dti-protocols/. The μCAPs analysis code is accessible at: https://github.com/MIPLabCH/mCAP.

## Figures and graphs

The brain networks were visualized with Mango image processing software (Lancaster, Martinez; www.ric.uthscsa.edu/mango). Graphs and figures were generated using Matlab©, Excel, PowerPoint, Biorender, and/or R©.

# SUPPLEMENTARY RESULTS AND DISCUSSION

## Details on the subgroups

Among the 97 EDD patients 30 were diagnosed with ADHD, 33 with BPD, and 34 with BD. Of the 34 patients with BD, 14 had BD type I, 15 had BD type II, and 5 unspecified BD. Among the 67 offspring of individuals with EDD (EDDoff), 23 had one parent affected by BD, 23 had one parent affected by BPD, and 21 had one parent affected by ADHD. To control for familial confounding factors, none of the participants across groups were biologically related. The vast majority of patients had recurrent episodes, and all were clinically stable at the time of assessment.

## Identification of the micro-coactivation patterns (μCAPs) and the corresponding hippocampal subdivisions

As previously described(1), the μCAPs analysis outlined in the Methods section led to the data-driven identification, across all participants, of five brain networks, each linked to a particular hippocampal subdivision (**Supplementary** **Figure 2**). The main μCAPs matched well-established resting-state networks from the literature(82–84), as follows. A sensorimotor-μCAP (SMN) predominantly covered pericentral and parietal regions, including the inferior parietal lobule, as well as auditory and visual areas. A limbic network-μCAP (LN) involved the orbitofrontal cortex, amygdala, insula, and ventral tegmental area (VTA). A default mode network-μCAP (DMN, also referred to as the medial frontoparietal network) included the posterior cingulate cortex, the angular gyrus, the precuneus, and the medial prefrontal cortex(82,83). The two remaining networks included a visual, occipital μCAP involving the superior colliculi, and a μCAP involving the VTA (**Supplementary Figure 2**).

Data-driven analysis of fMRI data indicated that, across all participants, each of these large-scale brain networks was associated with a data-driven hippocampal subdivision (**Supplementary Figure 2)**. The SMN was associated with the lateral body of the hippocampus. The LN, known for its involvement in emotion processing and regulation, was associated with the hippocampal head. The DMN was associated with the medial body of the hippocampus. Lastly, the visual network (VN) was associated with the hippocampal lateral tail, and the VTA with the hippocampal anterior body.

## Potential confounders

As detailed in **Table 1**, our analysis revealed no statistically significant differences among the three groups in terms of sex distribution, handedness, BMI, or cognitive scores, indicating overall well-balanced demographic and clinical characteristics across the cohorts.

To ensure that differences in hippocampal dFC were not influenced by participant motion during the fMRI scan, in addition to the motion adjustments described above, we conducted correlation analyses between FD and occurrences of hippocampal μCAPs. These analyses revealed no significant associations (*p* > 0.8 for all comparisons), thereby supporting the conclusion that observed μCAPs differences across groups were not confounded by subjects’ movement.

On the other hand, age significantly differed among groups, with EDDoff being predictably younger than the other two groups (*p* < 0.05 for all ANOVA post-hoc comparisons). However, several lines of evidence suggest that age did not substantially confound our main findings. Specifically, age was not prominently featured in any of the principal components associated with the primary outcomes reported in this study. Indeed, age was one of the primary contributors to only one retained PCA component (CL-RC3; **Supplementary Figure 14**), which was unrelated to the main findings reported in the article, showing only a modest negative correlation with a structural connectivity component (FA-RC1; **Supplementary Figure 15**), primarily characterized by FA in the SCR and PLIC regions, r(235) = −0.14, *p* = .038 (**Supplementary Figure 16**). This modest association likely reflects the well-established and documented decline of white matter integrity with increasing age(85).

Further corroborating the minimal role of age as a confounder, it was not ranked among the top 60% of most influential variables in any of the RF models predicting diagnostic group membership or ED symptoms. These results suggest that while age varied across groups, it was unlikely to have substantially confounded the primary outcomes of interest.

Lastly, as anticipated, approximately half of the EDD patients (49%) were prescribed psychotropic medication, contrary to the other groups. To evaluate whether psychotropic medication influenced the observed group differences, we computed a psychotropic treatment load index (0, 1, or 2 medications) for each patient. Correlation analyses (Spearman and Pearson) between this treatment load index and participants’ scores on retained PCA components showed no significant associations with any components exhibiting significant group differences (all *p* > 0.2), thereby supporting the conclusion that psychotropic medication use is unlikely to have driven the observed differences.

Collectively, these results suggest that demographic factors, such as age, as well as clinical factors, such as psychotropic medication usage, did not meaningfully bias or confound the findings presented in this study.

## Baseline linear model (ridge regression) for group prediction

Ridge regression achieved a test set classification accuracy of 74.3% (95% CI: 62.4%–84.0%) and a balanced accuracy (i.e. performance adjusted for group sizes) of 62.6%, with the optimal regularization parameter selected via 10-fold cross-validation. However, this performance was not significantly better than chance (No Information Rate = 70%; *p* = .26). The Cohen’s kappa coefficient was 0.29, indicating fair agreement between predicted and actual class labels(86).

Overall, these results highlight the limited ability of linear models to capture the complexity of group distinctions in this multimodal dataset, particularly in differentiating HC from at-risk or affected individuals.

## Negative control features confirm RF models validity

To assess the specificity and robustness of the RF models and the risk of overfitting, three synthetic noise features (Gaussian, random, and binomial) were included as negative controls in all classification and regression analyses.

Importantly, none of these noise features ranked among the top 60% of features in any RF model’s variable importance rankings. This pattern was consistent across both group classification and symptom prediction tasks. The lack of predictive value for these randomly generated features supports the specificity of the RF models and reduces the likelihood of overfitting or spurious associations.

These results strengthen confidence that the model’s predictive performance is driven by meaningful, biologically and clinically relevant variables rather than by noise or random artifacts.

## RF prediction of cognitive emotion regulation strategies

A RF regression model was trained to predict participants’ CERQ, using multimodal biological and neuroimaging variables. The model yielded a predictive performance of RMSE = 0.082, MAE = 0.065, and R² = 0.054. Despite the low explained variance, the prediction error remained relatively constrained, and actual and predicted CERQ scores showed a statistically significant correlation across participants (r = .32, *p* = .007, **Supplementary Figure 12**).

The most informative predictors included white matter integrity indices—particularly within the PCR and GCC- along with hippocampal dFC with the DMN. Interestingly, the proinflammatory cytokines IL-4 and MIF, and the anti-inflammatory cytokine IL-2 were the biological markers most relevant to the model (**Supplementary Figure 13**).

These results indicate that although the model's effect size was modest, immune and neuroimaging features contribute meaningfully to the prediction of individual variability in cognitive emotion regulation strategies.

## Group differences in the within-block PCA

Within-block PCA revealed group differences in a restricted set of components. In the immune-biological (BIO) block, BIO-RC3 (**Supplementary Figure 23)** showed a significant main effect of group (F(2,234) = 4.88, p = .008), with EDD patients scoring higher than both EDDoff (Tukey *p_adj_* = .022) and HC (*p_adj_* = .028), whereas EDDoff and HC did not differ (*p_adj_* = .99). In the structural FA block, RC2 differed across groups (F(2,234) = 3.11, p = .047), driven by lower scores in EDD relative to HC (*p_adj_* = .037); EDDoff did not differ significantly from either EDD or HC (both *p_adj_* ≥ .27). In the functional CAP block, dFC-RC1 (**Supplementary Figure 17)** showed a significant group effect (F(2,234) = 4.67, p = .010), with higher scores in EDD patients than in HC (*p_adj_* = .008), while EDDoff again did not differ from either group (*p_adj_* ≥ .11). In the clinical block, CL-RC1 (**Supplementary Figure 7**) showed a robust main effect of group (F(2,234) = 56.81, p < .001), with a clear gradient EDD > EDDoff > HC: EDD patients scored higher than both EDDoff and HC (both *p_adj_* < 1×10⁻⁷), and EDDoff scored higher than HC (*p_adj_* < .001). CL-RC3 also differed significantly between groups (F(2,234) = 9.77, p < .001): EDD patients had higher scores than EDDoff (*p_adj_* < .001), and HC scored higher than EDDoff (*p_adj_* = .0013), whereas EDD and HC did not differ (*p_adj_* = .93). No other pairwise group differences for any intra-modality component survived Tukey-adjusted post-hoc testing. These results are overall coherent with the multimodal results, which are reported in the main text. Detailed variable loadings and description for all retained components from the unimodal (clinical, FA, dFC, and biological) and multimodal PCAs are provided in **Supplementary Figures 16–29**, and discussed in the sections below.

## Multimodal diagnostic subgroup differences

One-way ANOVAs revealed significant subgroup diagnostic differences for the multimodal PCA components RC3, labeled “High emotion dysregulation–hippocampal dFC” (F(6,230) = 34.44, p < .001) and RC5, labeled “High Immune Dysregulation” (F(6,230) = 7.36, p < .001), consistent in direction with the transdiagnostic analyses reported in the main text. For RC3, post-hoc Tukey HSD comparisons indicated that all clinical groups showed significantly higher scores than controls (all *p_adj_* ≤ .006), with BPD patients exhibiting the highest scores and the largest effect relative to controls (*p_adj_* < .001). BPD scores were also significantly higher than those of BD (*p_adj_* < .001), BDOFF (*p_adj_* < .001), BPDOFF (*p_adj_* < .001), ADHD (p < .001), and ADHDOFF (*p_adj_* < .001, **Supplementary Figure 4**). For RC5, all patients’ diagnostic subgroups showed trends towards higher scores, although only the BD subgroup difference remained significant after multiple comparison adjustement (*p_adj_* < .001). No significant omnibus effects or pairwise differences surviving Tukey correction were observed for RC1, RC2, RC4, RC6, RC7, or RC8 (all p > .05). Given the modest size of each diagnostic subgroup, they should be interpreted as descriptive and underpowered rather than as definitive disorder-specific tests.

## Correlations among the retained components of multimodal PCA

Because the multimodal PCA was computed with an orthogonal Varimax rotation, the retained components are expected to be approximately uncorrelated at the group level. Nevertheless, as an empirical sanity check, we examined whether the two multimodal components that showed significant group differences (**Figures 2a–b**) were correlated at the subject level. Pearson’s and Spearman’s correlations between these two component scores did not reach statistical significance (p>0.8 for both analyses), indicating that the immune-related and ED/hippocampal connectivity components capture largely independent sources of variability. A heatmap summarizing all inter-block RC correlations is provided in **Supplementary Figure 31.**

## Further details on retained components of within-block PCAs

This section describes and discusses the retained components of within-block PCAs, which are further detailed in **Supplementary Figures 16-23**.

Within the white-matter integrity (FA) block, the first retained component (FA-RC1) explained 75.2% of the variance and reflected a global FA dimension across widespread projection and association tracts. Loadings were strongly positive in the corona radiata (SCR, PCR, ACR), internal capsule segments (PLIC, ALIC, IC, RLIC), superior longitudinal fasciculus (SLF), with a relative negative loading for the fornix (FX). Thus, higher FA-RC1 scores index overall higher white-matter integrity in these major pathways, with comparatively lower FA in the fornix. The second component (FA-RC2), which accounted for an additional 14.1% of variance, captured a more specific “callosal–cingulo” pattern: it showed high loadings in the body, genu, and splenium of the corpus callosum (BCC, GCC, CC) and cingulum bundle (CGC) and ACR. FA-RC3, as described in the main text, contributed 10.7% of white-matter variance, and captured FA concentrated in limbic and posterior association pathways, with highest loadings in the hippocampal cingulum (CGH), sagittal stratum (SS), external capsule (EC), retrolenticular internal capsule (RLIC), posterior thalamic radiation (PTR), and fornix-stria terminalis (FXST), consistent with a temporo-limbic/occipito-parietal microstructural axis.

In the clinical block, CL-RC1 (**Supplementary Figure 7**) explained 61.8% of the variance and represented a core ED/psychopathology factor: it loaded highly on trait anxiety (STAI-B), affective lability (ALS), borderline traits (BORD), non-adaptive emotion regulation (CERQ), ADHD symptoms (ASRS-i/hi), anger (STAXI), depressive symptoms (MADRS), rumination (RRS) and childhood trauma (CTQ), with small or negative loadings on working-memory and mental-calculation measures (IncrMem, RevDS, DigSpan, MenCal), indicating that more severe ED-related psychopathology tended to co-occur with slightly poorer cognitive performance. The second retained component (CL-RC2; 23.2% of explained variance) primarily indexed cognitive efficiency. It showed the highest positive loadings on incremental memory (IncrMem), backward digit span (RevDS), forward digit span (DigSpan), and mental calculation (MenCal), with smaller positive contributions from age, indicating that higher scores reflected better working memory and attentional control. In contrast, affective lability (ALS) and childhood trauma (CTQ) loaded negatively on this dimension, as did indices of inattention and hyperactivity (ASRSi/ASRShi), anxiety (STAIB), and BMI, suggesting that, also in this component, poorer cognitive performance tended to co-occur with higher emotional instability and subclinical psychopathology. The third component (CL-RC3; 15% of explained variance) captured a distinct “age–somatic load” and trauma dimension: it was dominated by positive loadings on age and BMI, as well as moderate positive loadings on CTQ, depressive symptoms (MADRS), and cognitive performance measures (DigSpan, RevDS, IncrMem), while ADHD symptoms (ASRSi/ASRShi) and borderline traits (BORD), along with ALS and anxiety, contributed negatively. Overall, CL-RC1 and CL-RC2 may reflect a gradient from lower to higher cognitive control and memory capacity in the context of ED and early life adversities, whereas CL-RC3 maps onto an axis combining older age and higher somatic burden with greater early adversity and mood symptoms but relatively fewer ADHD-like features.

The first and only principal component from the hippocampal micro-co-activation pattern block (dFC-RC1) included all five μCAPs (SMN, VTA, DMN, VN, LN), with the SMN as the only negative loading.

In the immunobiological blocks, as mentioned in the main text, BIO-RC1 (explaining 46.3% of the variance), reflected a broad immune dysregulation profile, with strong positive loadings on IL-12, IFN-γ, IL-17, IL-1β, TNF-α, IL-18, IFN-α, EGF and GM-CSF, and weaker negative loadings on adhesion and endothelial markers such as CD62E, VCAM, IL-6, GRO-α and IL-2; this axis therefore may capture a shift toward heightened Th1/Th17 and innate inflammatory signalling relative to vascular/endothelial activation. The second and third retained components captured two complementary axes of coordinated immune variation. The second component (BIO-RC2), which explained 21.7% of the total variance, showed strong positive loadings on canonical pro-inflammatory and growth-related markers, including IL-2, IL-6, GM-CSF, IL-8, type I interferons (IFNα, IFNβ), TNFα, IL-1β, MCP-1, IFNγ, IL-4 and EGF, and negative loadings on MIF, IL-18, IL-17, GROα, VCAM, CRP, BDNF, IL-12, IL-2R, LAP, IP-10, CD62E and IL-1α. Higher BIO-RC2 scores therefore index a profile dominated by IL-2/IL-6/GM-CSF/IL-8 and interferon activity, with relatively lower levels of adhesion, chemotactic and acute-phase markers. The third component (BIO-RC3), accounting for an additional 15.0% of variance, showed the opposite pattern for a partially distinct set of variables: it loaded positively on BDNF, IL-2R, MIF, MCP-1, CD62E, EGF, IL-18, CRP and IL-17, while exhibiting negative loadings on TNFα, IL-4, IL-1β, IL-8, IL-1α, GROα, IFNγ, IFNα, IFNβ, IL-6, IL-12, VCAM, IL-10, IL-2, GM-CSF, LAP and IP-10. As such, higher BIO-RC3 scores reflect a pattern of increased neurotrophic (BDNF), chemotactic (MCP-1), endothelial/adhesion (CD62E) and acute-phase/Th17-related markers (CRP, IL-17, IL-18), coupled with relatively reduced levels of several classical pro-inflammatory cytokines and growth factors. Together, BIO-RC2 and BIO-RC3 summarise distinct immune configurations, capturing coordinated shifts across multiple immune pathways rather than isolated changes in single analytes. Finally, BIO-RC4 accounted for a further 16.9% of immune variance and was dominated by chemokines and adhesion molecules (IP-10, LAP, GRO-α, VCAM) together with IL-17/IL-18 and soluble IL-2 receptor, suggesting a partially independent “chemokine/vascular activation” dimension of immune dysregulation.

## Further details on retained components of the multimodal PCA

This section describes and discusses the retained components of multimodal PCA, which are further detailed in **Supplementary Figures 24-29.**

As mentioned in the main text, in the combined multimodal PCA, RC3 explained 15.4% of the total variance and recapitulated the “Elevated ED–SMN Hypoconnectivity–LN Hyperconnectivity” pattern: it was dominated by the EDD-related psychopathology measures (STAI-B, ALS, BORD, ASRS-hi/-i, CERQ, STAXI, MADRS, RRS, CTQ) together with reduced hippocampal–sensorimotor (SMN) and increased hippocampal–limbic (LN) connectivity, and modest contributions from association-tract FA, thus linking behavioral ED severity to a coherent hippocampal dFC signature, as discussed in the main text. RC5, labeled “High Immune Dysregulation” (accounting for 7% of the multimodal variance), was instead primarily immunobiological, with strong positive loadings on BDNF, MIF, soluble IL-2 receptor, MCP-1, CD62E, EGF, CRP and IL-18 (and only minor contributions from imaging and clinical variables), indicating a largely orthogonal “neurotrophic–Immune Dysregulation” axis that cut across modalities but was less tightly coupled to overt ED symptomatology than RC3.

Several components other than the ED–hippocampal RC3 and the “High Immune Dysregulation” RC5 captured meaningful cross-domain structure. RC1 accounted for 28.8% of the total variance and was dominated by high positive loadings on fractional anisotropy in almost all major white-matter tracts (e.g. corona radiata, corpus callosum, internal capsule, association and projection fibres), with only minimal contribution from clinical, dFC or immune variables; this component therefore indexes a global white-matter integrity factor. RC2 explained a further 19.8% of the variance and was primarily an immune–inflammatory dimension, with strong positive loadings on pro-inflammatory cytokines and growth factors (IL-12, IL-4, IFN-γ, IL-10, IL-1β, IL-17, TNF-α, IFN-α, GM-CSF, IL-8, IL-6, VCAM and related markers), and smaller contributions from limbic/default-mode μCAPs, suggesting a systemic immune activation axis partly linked to large-scale network dynamics. RC4 (9.7% explained variance) also reflected immune activation, characterized by elevated loadings on IL-2, IL-6, GM-CSF, IL-8, interferons, MCP-1, MIF and CRP, representing a broad mixed T-cell/monocyte inflammatory profile mostly independent from other modalities. RC6, explaining 7.7% of the variance, captured a chemokine–endothelial/vascular component (IP-10, LAP, GRO-α, VCAM, IL-17, IL-18) coupled with FA in cortico-striatal tracts (e.g. CST, PLIC) and DMN/VTA connectivity. RC7 (5.9% variance) loaded most strongly on better memory and working-memory performance (Incremental Memory, Reverse Digit Span, Digit Span, Mental Calculation), and inversely with variables such as CRP and childhood trauma (CTQ). Finally, RC8 accounted for 5.6% of the variance and showed prominent (mainly negative) loadings on FA in the posterior and anterior limbs of the internal capsule, fornix and superior corona radiata, along with altered SMN/DMN μCAPs and only modest clinical/immune weights.

# SUPPLEMENTARY TABLES AND SUPPLEMENTARY TABLES LEGENDS

**Supplementary Table 1.** **Comprehensive list of all multimodal variables included in the analyses, along with their abbreviations.** Variables are grouped into four blocks: Clinical (CL), Hippocampal Dynamic Functional Connectivity (dFC), Structural Connectivity (FA), and Biological Markers (BIO). Variables are listed in alphabetical order within each block.

| **ABBREVIATION** | **DESCRIPTION** |
| --- | --- |
| **Clinical measures** | |
| Age | Chronological age in years |
| ALS | Affective Lability Scale total score |
| ASRShi | Adult ADHD Self-Report Scale - hyperactivity |
| ASRSi | Adult ADHD Self-Report Scale - inattention |
| BMI | Body Mass Index |
| BORD | Borderline Symptoms Checklist total score |
| CERQ | Cognitive Emotion Regulation Questionnaire  (Non-adaptive/Total) |
| CTQ | Childhood Trauma Questionnaire total score |
| DigSpan | Digit Span |
| IncrMem | Incremental Memory Task |
| MADRS | Montgomery–Åsberg Depression Rating Scale total score |
| MenCal | Mental Calculation |
| RevDS | Reverse Digit Span |
| RRS | Ruminative Response Scale total score |
| STAI-B | Trait Anxiety Inventory total score |
| STAXI | Trait Anger Expression Inventory total score |
| TEMPS | Temperament Evaluation of Memphis, Pisa, Paris, and San Diego scale total score |
| **Micro-CoActivation Patterns (μCAPs) hippocampal dynamic functional connectivity (dFC)** | |
| DMN | Default Mode Network dFC with the hippocampal medial body |
| LN | Limbic Network dFC with the hippocampal head |
| SMN | Sensorimotor Network dFC with hippocampal lateral body |
| VN | Visual Network dFC with the hippocampal lateral tail |
| VTA | Ventral Tegmental Area dFC with the hippocampal anterior body |
| **Fractional anisotropy (FA)** | |
| ACR | Anterior Corona Radiata FA |
| ALIC | Anterior Limb of Internal Capsule FA |
| BCC | Body of the Corpus Callosum FA |
| CC | Corpus Callosum FA |
| CGC | Cingulum (cingulate gyrus) FA |
| CGH | Perihippocampal cingulum tract FA |
| CR | Corona Radiata FA |
| CST | Corticospinal Tract FA |
| EC | External Capsule FA |
| FX | Fornix FA |
| FXST | Fornix Stria Terminalis FA |
| GCC | Genu of the Corpus Callosum FA |
| IC | Internal Capsule FA |
| PCR | Posterior Corona Radiata FA |
| PLIC | Posterior Limb of Internal Capsule FA |
| PTR | Posterior Thalamic Radiation FA |
| RLIC | Retrolenticular part of Internal Capsule FA |
| SCC | Splenium of the Corpus Callosum FA |
| SCR | Superior Corona Radiata FA |
| SFO | Superior Fronto‑Occipital fasciculus FA |
| SLF | Superior Longitudinal fasciculus FA |
| SS | Sagittal Stratum FA  (including parts of the inferior fronto-occipital fasciculus) |
| TP | Tapetum FA |
| UNC | Uncinate FA |
| **Biological markers (serum concentrations)** | |
| BDNF | Brain‑Derived Neurotrophic Factor |
| CD62E | E‑selectin |
| CRP | C-Reactive Protein |
| EGF | Epidermal Growth Factor |
| GM_CSF | Granulocyte–Macrophage Colony‑Stimulating Factor |
| GROa | Growth Regulated Protein-alpha/ CXCL1 |
| IFNa | Interferon‑alpha |
| IFNb | Interferon‑beta |
| IFNg | Interferon‑gamma |
| IL10 | Interleukin‑10 |
| IL12 | Interleukin‑12 |
| IL17 | Interleukin‑17a |
| IL18 | Interleukin‑18 |
| IL1a | Interleukin‑1 alpha |
| IL1b | Interleukin‑1 beta |
| IL2 | Interleukin‑2 |
| IL2R | Interleukin‑2 receptor |
| IL4 | Interleukin‑4 |
| IL6 | Interleukin‑6 |
| IL8 | Interleukin‑8 |
| IP10 | Interferon gamma inducible protein-10 |
| LAP | Latency-associated peptide |
| MCP1 | Monocyte Chemoattractant Protein‑1 |
| MIF | Macrophage Migration Inhibitory Factor |
| TNFa | Tumor Necrosis Factor‑alpha |
| VCAM | Vascular cell adhesion molecule 1 |

# SUPPLEMENTARY FIGURES AND SUPPLEMENTARY FIGURES LEGENDS

**Supplementary Figure 1.** **Theoretical framework recapitulating the links between inflammation, stress, emotion regulation networks, and clinical symptoms.** Emotion dysregulation (ED) symptoms may lead to higher chronic stress, which are in turn associated with a proinflammatory state. Neurotoxic effects of inflammation can negatively impact the neural circuits of emotion regulation, exacerbating symptoms. Thus, although the direction of potential causality between these elements remains to be ascertained, it appears likely that a vicious cycle perpetuating inflammation, central nervous system dysfunction, chronic stress, and ED symptoms may exist. Adapted with permission from(22).

**Supplementary Figure 2.** **Brain networks and related hippocampus subdivisions.** Micro-coactivation patterns (μCAPs) analysis allowed for the data-driven identification of five brain networks in all individuals, each of which was associated with a distinct subdivision based on hippocampus (HIP) activity. The hippocampus's lateral body, or L-body, was linked to a visual-sensorimotor μCAP (SMN). The hippocampus head was linked to the limbic network μCAP (LN). A default network μCAP (DMN) was linked to the hippocampal medial body (M-body). The lateral tail of the hippocampus was linked to a visual occipital μCAP. Lastly, the hippocampus anterior body (A-body) was linked to a μCAP involving the ventral tegmental region (VTA). Adapted with permission from (1).

****Supplementary Figure 3. Variable loadings on the “Elevated ED–SMN Hypoconnectivity–LN Hyperconnectivity” third retained principal component (RC3) from the PCA performed on the full multimodal dataset.**** Horizontal bars show each variable’s loading on RC3 (blue), with bars extending to the right indicating positive loadings and those extending to the left indicating negative loadings. Variables are sorted by the absolute magnitude of their loading, i.e. how strongly that variable contributes to RC3. Variables with large‐magnitude loadings (whether positive or negative) play a more important role in defining the component, while those with loadings near zero contribute minimally. This component is characterized by high positive loadings in ED symptoms such as non-adaptive emotion regulation strategies (CERQ), trait anxiety (STAI-B), affect lability (ALS), borderline symptoms (BORD) and ADHD hyperactivity (ASRShi), high positive loadings in hippocampal connectivity with the limbic network (LN), whereas measures such as sensorimotor network connectivity (SMN) load negatively. Please refer to Supplementary Table 1 for the complete list of all variables and corresponding abbreviations.

**Supplementary Figure 4. Distribution of RC3 scores across diagnostic groups.** Violin plots overlaid with boxplots display the distribution of individual scores on retained rotated component 3 (RC3) of the multimodal PCA, across the seven diagnostic groups. Horizontal lines within boxes indicate the median; boxes span the interquartile range. Group differences were assessed by one-way ANOVA followed by Tukey HSD post-hoc correction, and were coherent with the transdiagnostic findings reported in the main text for all diagnostic groups, as further described in the Supplementary Results.

****Supplementary Figure 5. Variable loadings on the third structural connectivity retained component (FA-RC3).****

Horizontal bars show each variable’s standardized loading on FA-RC3, sorted by absolute magnitude. Bars extending to the right indicate positive loadings. Please refer to Supplementary Table 1 for the complete list of all variables and corresponding abbreviations.

Supplementary ****Figure 6. Variable loadings on the first biological component (BIO-RC1).****
Horizontal bars show each variable’s standardized loading on BIO-RC1, sorted by absolute magnitude. Bars extending to the right indicate positive loadings, whereas bars extending to the left reflect negative loading. Please refer to Supplementary Table 1 for the complete list of all variables and corresponding abbreviations.

Supplementary Figure 7. ****Variable loadings on the first clinical component (CL-RC1).****
Horizontal bars show each variable’s standardized loading on CL-RC1, sorted by absolute magnitude. Bars extending to the right indicate positive loadings, whereas bars extending to the left reflect negative loading. Please refer to Supplementary Table 1 for the complete list of all variables and corresponding abbreviations.

Supplementary ****Figure 8. Variable loadings on the fourth biological component (BIO-RC4).****
Horizontal bars show each variable’s standardized loading on BIO-RC4, sorted by absolute magnitude. Bars extending to the right indicate positive loadings, whereas bars extending to the left reflect negative loading. Please refer to Supplementary Table 1 for the complete list of all variables and corresponding abbreviations.

**Supplementary Figure 9. Heatmap of the confusion matrix from Random Forest group classification, illustrating prediction accuracy between two participant groups: patients with Emotion Dysregulation Disorders (EDD) or EDD patients’ offspring (EDDoff) and healthy controls (HC).** Values in each cell represent the absolute number of predictions, with percentages indicating the proportion of each actual group's predictions. Color intensity corresponds to prediction frequency (Freq), with darker shades representing higher frequencies.

**Supplementary Figure 10. Variable importance in random forest classification model for group prediction.**

All variables are ranked by decrease in accuracy. This measure reflects how much the model's performance suffers when a particular feature is not available, indicating their contribution to accurate group classification. Clinical symptom measures of emotion dysregulation (e.g., ALS, BORD, MADRS, STAI-B) were among the strongest predictors, followed by neuroimaging-derived connectivity metrics. Immune biomarkers (e.g., CRP, IL-2R, MCP-1) showed relatively low importance, suggesting they contributed less to the model’s predictive performance. Please refer to Supplementary Table 1 for the complete list of all variables and corresponding abbreviations.

**Supplementary Figure 11. Correlation between actual and predicted affective lability scale (ALS) scores.** Scatterplot of observed versus RF‐predicted ALS scores in the held‑out test, from neuroimaging and biological features, with a linear fit overlaid. The overall Pearson correlation coefficient (r =.48, p*_adj_* < .00005), indicates robust, transdiagnostic convergence between model prediction and actual affective lability severity.

 **Supplementary Figure 12. Correlation between actual and predicted Cognitive Emotion Regulation Questionnaire (CERQ) scores.** Scatterplot of observed versus RF‐predicted CERQ scores in the held‑out test, with a linear fit overlaid. The overall significant positive correlation (p*_adj_* = .007) indicates transdiagnostic convergence between predicted and actual CERQ scores.

Supplementary Figure 13. Feature importance for predicting Cognitive Emotion Regulation Questionnaire (CERQ) scores from neuroimaging and biological features.

Variables are ranked by importance (measured as percentage of increase in Mean Squared Error with permutation, %IncMSE) from the random forest regression model predicting CERQ scores. Higher %IncMSE values indicate greater predictive contribution. Dot color coding reflects Spearman correlation (ρ) between each feature and CERQ scores, blue for positive and red for negative associations. Please refer to Supplementary Table 1 for the complete list of all variables and corresponding abbreviations.

**Supplementary Figure 14. Variable loadings for the third retained component from the clinical block (CL-RC3).** CL-RC3 reflects an additional, partially independent clinical pattern, combining mainly age and BMI, along with aspects of mood, anxiety, emotion dysregulation, cognition, and demographic measures. The plot reports rotated loadings for each clinical variable, illustrating which measures contribute most strongly and in which direction to individual CL-RC3 scores.

****Supplementary Figure 15. Variable loadings for the first retained component from the white-matter integrity block (FA-RC1).** This component describes a pattern of fractional anisotropy variation across major association, projection, and commissural tracts (e.g., corona radiata, internal capsule, …). The bar plot depicts rotated loadings for each tract-specific FA measure, with larger absolute indicating tracts that contribute most prominently to FA-RC1.**

**Supplementary Figure 16. Scatter plot showing the relationship between CL-RC3 (Clinical–Retained Component 3) and FA-RC1 (Fractional Anisotropy Retained Component 1) across groups (EDD, EDDoff, HC).** A transdiagnostic, significant negative correlation was observed between the age-driven clinical component (CL-RC3) and the structural connectivity component FA-RC1, indicating that some variability in lower white matter integrity across widespread tracts may be associated with higher age.

**Supplementary Figure 17.** **Variable loadings for the first and only principal component from the hippocampal micro-co-activation pattern block (dFC-RC1).** The bar plot shows the rotated loadings of each μCAP state (SMN, VTA, DMN, VN, LN) on dFC-RC1. Higher absolute loadings indicate a stronger contribution of that μCAP state to the component score, with positive and negative (e.g. SMN) bars reflecting μCAP states that vary in opposite directions along this functional connectivity dimension.

**Supplementary Figure 18.** **Variable loadings for the second retained component from the clinical block (CL-RC2).** This component captures a distinct clinical dimension summarizing shared variance across affective symptoms, emotion regulation indices, trauma exposure, neurocognitive measures, and BMI. Bars represent rotated factor loadings for each clinical variable; higher absolute values indicate stronger contribution to CL-RC2, with opposite signs marking variables that load in contrasting directions on this dimension.

**Supplementary Figure 19. Variable loadings for the third retained component from the clinical block (CL-RC3).** CL-RC3 reflects an additional, partially independent clinical pattern, combining mainly age and BMI, along with aspects of mood, anxiety, emotion dysregulation, cognition, and demographic measures. The plot reports rotated loadings for each clinical variable, illustrating which measures contribute most strongly and in which direction to individual CL-RC3 scores.

**Supplementary Figure 20.** **Variable loadings for the first retained component from the white-matter integrity block (FA-RC1).** This component describes a pattern of fractional anisotropy variation across major association, projection, and commissural tracts (e.g., corona radiata, internal capsule, …). The bar plot depicts rotated loadings for each tract-specific FA measure, with larger absolute indicating tracts that contribute most prominently to FA-RC1.

**Supplementary Figure 21.** **Variable loadings for the second retained component from the white-matter integrity block (FA-RC2).** This component describes a secondary pattern of fractional anisotropy variation across major association, projection, and commissural tracts (e.g., corpus callosum segments, internal capsule, corona radiata…). The bar plot depicts rotated loadings for each tract-specific FA measure, with larger absolute indicating tracts that contribute most prominently to FA-RC2.

**Supplementary Figure 22.** **Variable loadings for the second retained component from the immunobiological block based on raw serum markers (BIO-RC2).** This component summarizes shared variance across subsets of cytokines, chemokines, growth factors, and adhesion molecules (e.g., interleukins, TNFα, CRP, VCAM). Rotated loadings are shown for each biomarker, highlighting those that contribute most to BIO-RC2 and the direction of their association with the component scores.

**Supplementary Figure 23.** **Variable loadings for the third retained component from the immunobiological block based on raw serum markers (BIO-RC3).** BIO-RC3 represents an additional, independent axis of immune variation across the measured neurogenesis (i.e. BDNF) and immune markers. The bar plot shows rotated loadings for each biomarker, indicating which analytes co-vary along this component and distinguishing markers with positive versus negative contributions.

**Supplementary Figure 24.** **Variable loadings for the first retained component from the full multimodal PCA (RC1-FullPCA).** This component captures a global pattern of shared variance spanning clinical, white-matter, functional connectivity, and immunobiological measures. Loadings are plotted for all variables included in the multimodal analysis, grouped by modality, illustrating which features contribute most strongly (in absolute value) to this overarching multimodal dimension.

**Supplementary Figure 25.** **Variable loadings for the second retained component from the full multimodal PCA (RC2-FullPCA).** RC2-FullPCA reflects a second, independent multimodal pattern that differentiates subsets of clinical, imaging, and biological variables. The bar plot displays rotated loadings for each variable, allowing visual inspection of modality-specific and cross-modal contributions to this component.

**Supplementary Figure 26.** **Variable loadings for the fourth retained component from the full multimodal PCA (RC4-FullPCA).** This figure shows the rotated factor loadings of all clinical, FA, CAP, and biological variables on RC4-FullPCA, highlighting a more specific cross-domain pattern that explains additional variance beyond the first three multimodal components.

**Supplementary Figure 27.** **Variable loadings for the sixth retained component from the full multimodal PCA (RC6-FullPCA).** RC6-FullPCA represents a higher-order multimodal dimension capturing relatively subtle but distinct co-variation among a subset of clinical, neuroimaging, and immune features. Bars indicate the size and sign of the rotated loadings for each variable.

**Supplementary Figure 28.** **Variable loadings for the seventh retained component from the full multimodal PCA (RC7-FullPCA).** This component accounts for additional residual multimodal variance, with contributions from selected clinical, white-matter, functional, and serum markers. The loading plot illustrates which variables define RC7-FullPCA and whether they load positively or negatively on this dimension.

**Supplementary Figure 29.** **Variable loadings for the eighth retained component from the full multimodal PCA (RC8-FullPCA).** RC8-FullPCA captures a further, relatively low-variance multimodal pattern. The figure presents the rotated loadings for all included variables, provided for completeness to document the structure of all retained components in the multimodal PCA.

**Supplementary Figure 30. Illustration of the main JHU ROIs relevant to the analysis.** Locations of the ROIs from the JHU ICBM-DTI-81 white matter labels atlas are shown in the glass brain for each broad group: (A) projection, (B) association, and (C) commissural fibers. See **Supplementary Table** **1** for the full ROI names corresponding to the abbreviations in the figure. Figure adapted with written permission from Tsuchida et al. (2021)(87), licensed under CC BY 4.0.

**Supplementary Figure** 31**.** **Heatmap of Pearson correlations between retained principal components (RCs) derived from the block-wise PCA of each modality**. Clinical/behavioral (CL), hippocampal dynamic functional connectivity measured with micro-coactivation patterns, μCAPs (CAP), white-matter microstructure (FA), and biological markers (BIO). Rows and columns correspond to RCs from the different blocks; warmer colors indicate stronger positive correlations and cooler colors indicate stronger negative correlations (values close to zero are near-neutral).

# SUPPLEMENTARY REFERENCES

1. Saccaro LF, Delavari F, Meuleman B, Perroud N, Van De Ville D, Piguet C (2025): Limbic-Sensorimotor Tug of War for the Hippocampus: Dynamic Functional Connectivity as a Transdiagnostic Vulnerability Marker in Offspring of Emotion Dysregulation Patients. *Biol Psychiatry Cogn Neurosci Neuroimaging*. https://doi.org/10.1016/j.bpsc.2025.03.007

2. Ellison WD, Rosenstein LK, Morgan TA, Zimmerman M (2018): Community and Clinical Epidemiology of Borderline Personality Disorder. *Psychiatr Clin North Am* 41: 561–573.

3. Collaborators GBD 2019 MD (2019): Global , regional , and national burden of 12 mental disorders in 204 countries and territories , 1990 – 2019 : a systematic analysis from the Global Burden of Disease Study 2019 Accepted for publication at The Lancet Psychiatry. *Lancet Psychiatry* 0366: 1–43.

4. Skoglund C, Tiger A, Rück C, Petrovic P, Asherson P, Hellner C, *et al.* (2021): Familial risk and heritability of diagnosed borderline personality disorder: a register study of the Swedish population. *Mol Psychiatry* 26: 999–1008.

5. Stahl EA, Breen G, Forstner AJ, McQuillin A, Ripke S, Trubetskoy V, *et al.* (2019): Genome-wide association study identifies 30 loci associated with bipolar disorder. *Nat Genet* 51: 793–803.

6. Lener MS (2016): Genome-wide analyses of borderline personality features. *Physiol Behav* 176: 139–148.

7. Ohi K, Nishizawa D, Shimada T, Kataoka Y, Hasegawa J, Shioiri T, *et al.* (2020): Polygenetic risk scores for major psychiatric disorders among schizophrenia patients, their first-degree relatives, and healthy participants. *Int J Neuropsychopharmacol* 23: 157–164.

8. Demontis D, Walters RK, Martin J, Mattheisen M, Als TD, Agerbo E, *et al.* (2019): Discovery of the first genome-wide significant risk loci for attention deficit/hyperactivity disorder. *Nat Genet* 51: 63–75.

9. Katzman MA, Bilkey TS, Chokka PR, Fallu A, Klassen LJ (2017): Adult ADHD and comorbid disorders: Clinical implications of a dimensional approach. *BMC Psychiatry* 17: 1–15.

10. Weiner L, Perroud N, Weibel S (2019): Attention Deficit Hyperactivity Disorder And Borderline Personality Disorder In Adults: A Review Of Their Links And Risks. *Neuropsychiatr Dis Treat* 15: 3115–3129.

11. Zimmerman M, Morgan TA (2013): The relationship between borderline personality disorder and bipolar disorder. *Dialogues Clin Neurosci* 15: 155.

12. Perroud N, Cordera P, Zimmermann J, Michalopoulos G, Bancila V, Prada P, *et al.* (2014): Comorbidity between attention deficit hyperactivity disorder (ADHD) and bipolar disorder in a specialized mood disorders outpatient clinic. *J Affect Disord* 168: 161–166.

13. Townsend JD, Sugar CA, Walshaw PD, Vasquez RE, Foland-Ross LC, Moody TD, *et al.* (2013): Frontostriatal neuroimaging findings differ in patients with bipolar disorder who have or do not have ADHD comorbidity. *J Affect Disord* 147: 389–396.

14. Asherson P, Young AH, Eich-Höchli D, Moran P, Porsdal V, Deberdt W (2014): Differential diagnosis, comorbidity, and treatment of attention-deficit/hyperactivity disorder in relation to bipolar disorder or borderline personality disorder in adults. *Curr Med Res Opin* 30: 1657–1672.

15. Richard-Lepouriel H, Etain B, Hasler R, Bellivier F, Gard S, Kahn J-P, *et al.* (2016): Similarities between emotional dysregulation in adults suffering from ADHD and bipolar patients. *J Affect Disord* 198: 230–236.

16. Arnold LE, Mount K, Frazier T, Demeter C, Youngstrom EA, Fristad MA, *et al.* (2012): Pediatric bipolar disorder and ADHD: family history comparison in the LAMS clinical sample. *J Affect Disord* 141: 382–389.

17. Linhartová P, Látalová A, Barteček R, ŠirÅček J, Theiner P, Ejova A, *et al.* (2019): Impulsivity in patients with borderline personality disorder: A comprehensive profile compared with healthy people and patients with ADHD. *Psychol Med*. https://doi.org/10.1017/S0033291719001892

18. Casey BJ, Heller AS, Gee DG, Cohen AO (2019): Development of the emotional brain. *Neurosci Lett* 693: 29–34.

19. Sanchis-Sanchis A, Grau MD, Moliner A-R, Morales-Murillo CP (2020): Effects of Age and Gender in Emotion Regulation of Children and Adolescents. *Front Psychol* 11: 946.

20. Childress AC, Stark JG (2018): Diagnosis and treatment of attention-deficit/hyperactivity disorder in preschool-aged children. *J Child Adolesc Psychopharmacol* 28: 606–614.

21. Saccaro LF, Giff A, De Rossi MM, Piguet C (2024): Interventions targeting emotion regulation: A systematic umbrella review. *J Psychiatr Res* 174: 263–274.

22. Petruso F, Giff AE, Milano BA, Menduni De Rossi M, Saccaro LF (2023): Inflammation and emotion regulation: a narrative review of evidence and mechanisms in emotion dysregulation disorders. *Neuronal Signal* NS20220077.

23. Faraone SV, Banaschewski T, Coghill D, Zheng Y, Biederman J, Bellgrove MA, *et al.* (2021): The World Federation of ADHD International Consensus Statement: 208 Evidence-based conclusions about the disorder. *Neurosci Biobehav Rev* 128: 789–818.

24. Merikangas KR, Jin R, He JP, Kessler RC, Lee S, Sampson NA, *et al.* (2011): Prevalence and correlates of bipolar spectrum disorder in the World Mental Health Survey Initiative. *Arch Gen Psychiatry* 68: 241–251.

25. Grant BF, Chou SP, Goldstein RB, Huang B, Stinson FS, Saha TD, *et al.* (2008): Prevalence, correlates, disability, and comorbidity of DSM-IV borderline personality disorder: results from the Wave 2 National Epidemiologic Survey on Alcohol and Related Conditions. *J Clin Psychiatry* 69: 533–545.

26. Eich D, Gamma A, Malti T, Vogt Wehrli M, Liebrenz M, Seifritz E, Modestin J (2014): Temperamental differences between bipolar disorder, borderline personality disorder, and attention deficit/hyperactivity disorder: some implications for their diagnostic validity. *J Affect Disord* 169: 101–104.

27. Kebets V, Favre P, Houenou J, Polosan M, Perroud N, Aubry JM, *et al.* (2021): Fronto-limbic neural variability as a transdiagnostic correlate of emotion dysregulation. *Transl Psychiatry* 11: 1–8.

28. Bayas M, Kockler TD, Ramos-Quiroga JA, Caller SM, Fadeuilhe C, de Girolamo G, *et al.* (2024): Dynamics of affect modulation in neurodevelopmental disorders (DynAMoND) – study design of a prospective cohort study. *Int J Bipolar Disord* 12: 44.

29. Durpoix A, Lachaux E, Weiner L, Weibel S (2023): Transdiagnostic skills training group of dialectical behavior therapy: a long-term naturalistic study. *Borderline Personal Disord Emot Dysregulation* 10: 37.

30. Phillips ML, Swartz HA (2014): A critical appraisal of neuroimaging studies of bipolar disorder: Toward a new conceptualization of underlying neural circuitry and a road map for future research. *Am J Psychiatry* 171: 829–843.

31. Engen HG, Anderson MC (2018): Memory Control: A Fundamental Mechanism of Emotion Regulation. *Trends Cogn Sci* 22: 982–995.

32. Genon S, Bernhardt BC, Joie RL, Amunts K, Eickhoff SB (2021): The many dimensions of human hippocampal organization and (dys)function. *Trends Neurosci* 44: 977–989.

33. Plachti A, Eickhoff SB, Hoffstaedter F, Patil KR, Laird AR, Fox PT, *et al.* (2019): Multimodal Parcellations and Extensive Behavioral Profiling Tackling the Hippocampus Gradient. *Cereb Cortex* 29: 4595–4612.

34. Ezama L, Hernández-Cabrera JA, Seoane S, Pereda E, Janssen N (2021): Functional connectivity of the hippocampus and its subfields in resting-state networks. *Eur J Neurosci* 53: 3378–3393.

35. Sala M, Perez J, Soloff P, Nemi SU di, Caverzasi E, Soares JC, Brambilla P (2004): Stress and hippocampal abnormalities in psychiatric disorders. *Eur Neuropsychopharmacol* 14: 393–405.

36. Baumann PS, Griffa A, Ferrari C, Alameda L, Thiran J-P, Do KQ, *et al.* (2014): Integrity of the fornix and hippocampus and relationship with peripheral oxidative stress markers in early phase psychosis. *Biol Psychiatry* 75: 109S.

37. Chesnokova V, Pechnick RN, Wawrowsky K (2016): Chronic peripheral inflammation, hippocampal neurogenesis, and behavior. *Brain Behav Immun* 58: 1–8.

38. Zonis S, Pechnick RN, Ljubimov VA, Mahgerefteh M, Wawrowsky K, Michelsen KS, Chesnokova V (2015): Chronic intestinal inflammation alters hippocampal neurogenesis. *J Neuroinflammation* 12: 1–12.

39. Kraus KL, Chordia AP, Drake AW, Herman JP, Danzer SC (2022): Hippocampal interneurons are direct targets for circulating glucocorticoids. *J Comp Neurol*.

40. Dedovic K, Duchesne A, Andrews J, Engert V, Pruessner JC (2009): The brain and the stress axis: The neural correlates of cortisol regulation in response to stress. *NeuroImage* 47: 864–871.

41. Gee DG, Gabard-Durnam LJ, Flannery J, Goff B, Humphreys KL, Telzer EH, *et al.* (2013): Early developmental emergence of human amygdala-prefrontal connectivity after maternal deprivation. *Proc Natl Acad Sci U S A* 110: 15638–15643.

42. Yuan JP, Ho TC, Coury SM, Chahal R, Colich NL, Gotlib IH (2022): Early life stress, systemic inflammation, and neural correlates of implicit emotion regulation in adolescents. *Brain Behav Immun* 105: 169–179.

43. Mathur A, Li JC, Lipitz SR, Graham-Engeland JE (2022): Emotion Regulation as a Pathway Connecting Early Life Adversity and Inflammation in Adulthood: a Conceptual Framework. *Advers Resil Sci* 3: 1–19.

44. Caruso D, Palagini L, Miniati M, Massa L, Marazziti D, Geoffroy PA, Etain B (2021): Early Life Stress and Chronobiological Rhythms Desynchronization: Possible Impact on Mood Symptoms and Suicidal Ideation in Bipolar Disorder. *J Nerv Ment Dis* 209. Retrieved from https://journals.lww.com/jonmd/Fulltext/2021/07000/Early_Life_Stress_and_Chronobiological_Rhythms.10.aspx

45. Saccaro LF, Schilliger Z, Perroud N, Piguet C (2021): Inflammation, Anxiety, and Stress in Attention-Deficit/Hyperactivity Disorder. *Biomedicines* 9: 1313.

46. Saccaro LF, Schilliger Z, Dayer A, Perroud N, Piguet C (2021): Inflammation, anxiety, and stress in bipolar disorder and borderline personality disorder: A narrative review. *Neurosci Biobehav Rev* 127: 184–192.

47. Iob E, Kirschbaum C, Steptoe A (2020): Persistent depressive symptoms, HPA-axis hyperactivity, and inflammation: the role of cognitive-affective and somatic symptoms. *Mol Psychiatry* 25: 1130–1140.

48. Chen X, Gianferante D, Hanlin L, Fiksdal A, Breines JG, Thoma MV, Rohleder N (2017): HPA-axis and inflammatory reactivity to acute stress is related with basal HPA-axis activity. *Psychoneuroendocrinology* 78: 168–176.

49. Tseng HH, Chang HH, Wei SY, Lu TH, Hsieh YT, Yang YK, Chen PS (2021): Peripheral inflammation is associated with dysfunctional corticostriatal circuitry and executive dysfunction in bipolar disorder patients. *Brain Behav Immun* 91: 695–702.

50. Tsai SY, Gildengers AG, Hsu JL, Chung KH, Chen PH, Huang YJ (2019): Inflammation associated with volume reduction in the gray matter and hippocampus of older patients with bipolar disorder. *J Affect Disord* 244: 60–66.

51. Saccaro LF, Gaviria J, Ville DVD, Piguet C (2023): Dynamic functional hippocampal markers of residual depressive symptoms in euthymic bipolar disorder. *Brain Behav* 13: e3010.

52. Saccaro LF, Delavari F, Van De Ville D, Piguet C (2024): Hippocampal temporal dynamics and spatial heterogeneity unveil vulnerability markers in the offspring of bipolar patients. *Bipolar Disord*. https://doi.org/10.1111/bdi.13487

53. Zhong S, Chen P, Lai S, Zhang Y, Chen G, He J, *et al.* (2024): Hippocampal Dynamic Functional Connectivity, HPA Axis Activity, and Personality Trait in Bipolar Disorder with Suicidal Attempt. *Neuroendocrinology* 114: 179–191.

54. Nurnberger JI, Blehar MC, Kaufmann CA, York-Cooler C, Simpson SG, Harkavy-Friedman J, *et al.* (1994): Diagnostic interview for genetic studies. Rationale, unique features, and training. NIMH Genetics Initiative. *Arch Gen Psychiatry* 51: 849–859; discussion 863-864.

55. Garnefski N, Kraaij V, Spinhoven P (2001): Negative life events, cognitive emotion regulation and emotional problems. *Personal Individ Differ* 30: 1311–1327.

56. Gadassi-Polack R, Paganini G, Zhang AK, Dworschak C, Silk JS, Kober H, Joormann J (2024): It’s a balancing act: The ratio of maladaptive (vs. All) emotion regulation strategies is associated with depression. *Behav Res Ther* 180: 104600.

57. Harvey PD, Greenberg BR, Serper MR (1989): The affective lability scales: Development, reliability, and validity. *J Clin Psychol* 45: 786–793.

58. Thornton OR, Li W, Cole H, Cólon I (2023): The Role of the Hippocampus in Borderline Personality Disorder: Structural and Functional Abnormalities. *Int Neuropsychiatr Dis J* 19: 28–38.

59. Zöller D, Schaer M, Scariati E, Padula MC, Eliez S, Ville DVD (2017): Disentangling resting-state BOLD variability and PCC functional connectivity in 22q11.2 deletion syndrome. *NeuroImage* 149: 85–97.

60. Ashburner J, Friston KJ (2005): Unified segmentation. *NeuroImage* 26: 839–851.

61. Ashburner J (2007): A fast diffeomorphic image registration algorithm. *NeuroImage* 38: 95–113.

62. Chao-Gan Y, Yu-Feng Z (2010): DPARSF: A MATLAB toolbox for “pipeline” data analysis of resting-state fMRI. *Front Syst Neurosci* 4. https://doi.org/10.3389/fnsys.2010.00013

63. Power JD, Barnes KA, Snyder AZ, Schlaggar BL, Petersen SE (2012): Spurious but systematic correlations in functional connectivity MRI networks arise from subject motion. *NeuroImage* 59: 2142–2154.

64. Robinson JL, Barron DS, Kirby LAJ, Bottenhorn KL, Hill AC, Murphy JE, *et al.* (2015): Neurofunctional topography of the human hippocampus. *Hum Brain Mapp* 36: 5018–5037.

65. Bolton TAW, Tuleasca C, Wotruba D, Rey G, Dhanis H, Gauthier B, *et al.* (2020): TbCAPs: A toolbox for co-activation pattern analysis. *NeuroImage* 211: 116621.

66. Delavari F, Sandini C, Kojovic N, Saccaro LF, Eliez S, Van De Ville D, Bolton TAW (2024): Thalamic contributions to psychosis susceptibility: Evidence from co-activation patterns accounting for intra-seed spatial variability (μCAPs). *Hum Brain Mapp* 45: e26649.

67. Liu X, Zhang N, Chang C, Duyn JH (2018): Co-activation patterns in resting-state fMRI signals. *NeuroImage* 180: 485–494.

68. Vidaurre D, Smith SM, Woolrich MW (2017): Brain network dynamics are hierarchically organized in time. *Proc Natl Acad Sci* 114: 12827–12832.

69. Rey G, Bolton TAW, Gaviria J, Piguet C, Preti MG, Favre S, *et al.* (2021): Dynamics of amygdala connectivity in bipolar disorders: A longitudinal study across mood states. *medRxiv* 2021.03.30.21254608.

70. Bolton TAW, Wotruba D, Buechler R, Theodoridou A, Michels L, Kollias S, *et al.* (2020): Triple Network Model Dynamically Revisited: Lower Salience Network State Switching in Pre-psychosis. *Front Physiol* 11: 1–10.

71. Delavari F, Sandini C, Kojovic N, Saccaro LF, Eliez S, Ville DVD, Bolton TAW (n.d.): Thalamic contributions to psychosis susceptibility: Evidence from co-activation patterns accounting for intra-seed spatial variability (µCAPs). https://doi.org/10.1101/2023.05.23.541896

72. Gaviria J, Rey G, Bolton T, Ville DVD, Vuilleumier P (2021): Dynamic functional brain networks underlying the temporal inertia of negative emotions. *NeuroImage* 240. https://doi.org/10.1016/j.neuroimage.2021.118377

73. Kuhn HW (1956): Variants of the hungarian method for assignment problems. *Nav Res Logist Q* 3: 253–258.

74. Smith S, Jenkinson M, Johansen-Berg H, Rueckert D, Nichols T, Mackay C, *et al.* (2006): Tract-based spatial statistics: Voxelwise analysis of multi-subject diffusion data. *NEUROIMAGE* 31: 1487–1505.

75. Buuren S van (2018): *Flexible Imputation of Missing Data, Second Edition*, 2nd ed. New York: Chapman and Hall/CRC. https://doi.org/10.1201/9780429492259

76. Hastie T, Tibshirani R, Friedman J (2009): Random Forests. In: Hastie T, Tibshirani R, Friedman J, editors. *The Elements of Statistical Learning: Data Mining, Inference, and Prediction*. New York, NY: Springer, pp 587–604.

77. Díaz-Uriarte R, Alvarez de Andrés S (2006): Gene selection and classification of microarray data using random forest. *BMC Bioinformatics* 7: 3.

78. Breiman L (2001): Random Forests. *Mach Learn* 45: 5–32.

79. Biau G (2010): Analysis of a Random Forests Model. *J Mach Learn Res* 13.

80. Liaw, A. and Wiener, M. (2002) Classification and Regression by Randomforest. R News, 2, 18-22. - References - Scientific Research Publishing (n.d.): Retrieved December 16, 2025, from https://www.scirp.org/reference/referencespapers?referenceid=2107686

81. Hyperparameters and tuning strategies for random forest - Probst - 2019 - WIREs Data Mining and Knowledge Discovery - Wiley Online Library (n.d.): Retrieved April 24, 2025, from https://wires.onlinelibrary.wiley.com/doi/10.1002/widm.1301

82. Uddin LQ, Yeo BTT, Spreng RN (2019): Towards a Universal Taxonomy of Macro-scale Functional Human Brain Networks. *Brain Topogr* 32: 926–942.

83. Gao W, Biswal B, Chen S, Wu X, Yuan J (2021): Functional coupling of the orbitofrontal cortex and the basolateral amygdala mediates the association between spontaneous reappraisal and emotional response. *NeuroImage* 232: 117918.

84. Han Z, Liu T, Shi Z, Zhang J, Suo D, Wang L, *et al.* (2023): Investigating the heterogeneity within the somatosensory-motor network and its relationship with the attention and default systems. *PNAS Nexus* 2: pgad276.

85. Grieve SM, Williams LM, Paul RH, Clark CR, Gordon E (2007): Cognitive Aging, Executive Function, and Fractional Anisotropy: A Diffusion Tensor MR Imaging Study. *AJNR Am J Neuroradiol* 28: 226–235.

86. McHugh ML (2012): Interrater reliability: the kappa statistic. *Biochem Medica* 22: 276–282.

87. Tsuchida A, Laurent A, Crivello F, Petit L, Pepe A, Beguedou N, *et al.* (2021): Age-Related Variations in Regional White Matter Volumetry and Microstructure During the Post-adolescence Period: A Cross-Sectional Study of a Cohort of 1,713 University Students. *Front Syst Neurosci* 15. https://doi.org/10.3389/fnsys.2021.692152
